# Diversity Patterns of Domestic Herbivore Viruses in China Reveal Transmission Dynamics with Disease Management Implications

**DOI:** 10.1002/advs.202517444

**Published:** 2026-03-25

**Authors:** Yue Sun, Yong Li, Bagen Temur, Yuanqing Lin, Yuhang Liu, Le Yi, Zheng Sun, Gang Zhang, Jun Li, Yu Guo, Linchuan Li, Jinshan Cai, Wenliang Tian, Gen Meng, Lingling Jiang, Min Fang, Fuying Ding, Xuezhang Zhou, Changchun Tu, Biao He

**Affiliations:** ^1^ State Key Laboratory of Pathogen and Biosecurity Changchun Veterinary Research Institute Chinese Academy of Agricultural Sciences Changchun Jilin China; ^2^ Key Laboratory of Ministry of Education for Protection and Utilization of Special Biological Resources in Western China School of Life Sciences Ningxia University Yinchuan China; ^3^ Inner Mongolia Animal Disease Prevention and Control Center Hohhot Inner Mongolia China; ^4^ Qinghai Provincial Animal Disease Prevention and Control Center Xining Qinghai China; ^5^ Siziwang Banner Animal Disease Prevention and Control Center Wulanchabu Inner Mongolia China; ^6^ Wulanchabu Animal Disease Prevention and Control Center Wulanchabu Inner Mongolia China; ^7^ Qilian County Animal Disease Prevention and Control Center Qilian Qinghai China; ^8^ Jiangsu Co‐Innovation Center for Prevention and Control of Important Animal Infectious Diseases and Zoonosis Yangzhou University Yangzhou Jiangsu China

**Keywords:** circulation dynamics, domestic herbivores, emerging infectious diseases, pan‐viromics, wildlife conservation

## Abstract

Domestic herbivores have complex interactions with humans and wildlife, playing important roles in zoonotic and epizootic disease emergence and transmission. Yet their viral diversity and cross‐species transmission dynamics remain understudied. Through pan‐viromic profiling of 10,225 swabs and 4,304 serum samples from 5,710 adult individuals across China's five major herbivore‐rearing provinces, we prepare the domestic herbivore viromic catalog of China (DhCN‐Virome) comprising 1,085,360 viral metagenomes, nearly capturing their family‐level viral diversity while expanding by 2.3‐fold global subgenus‐level viral diversity. Distinct viromic signatures emerge across herbivore species and sample types. Viral communities generally follow a “higher openness, greater stability” pattern, with animals raised in confined settings being more susceptible to external influences. Viral circulations, particularly involving viruses of health concern, occur primarily within herbivore species but also extensively between herbivores and other species, including potential human‐herbivore and avian‐horse viral transmission. Bacteriophages constitute the most abundant viral entities, characterized by lytic replication strategies with some targeting pathogenic bacterial hosts. These findings expand our knowledge of herbivore viral diversity patterns and ecological transmission dynamics, underscoring the need for unified disease management strategies across all herbivore species. Particularly, the risk viruses represent potential triggers for future outbreaks, necessitating urgent epidemiological surveillance and vaccination programs.

## Graphical abstract

This study performs pan‐viromic profiling of 14,529 samples from 5,710 domestic herbivores across five Chinese provinces, establishing the DhCN‐Virome (1,085,360 viral metagenomes). It reveals species/sample‐specific viromic signatures and cross‐species transmission dynamics, aiding unified disease control.

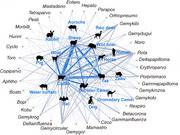

## 1 Introduction

Viral cross‐species transmission can trigger zoonotic emergences and drive epizootic outbreaks [1]. Deciphering the global viral diversity of key animal groups and mapping their cross‐species transmission networks provides a critical framework for mitigating and preventing future infectious disease crises [2, 3]. Domestic herbivores sustain intricate biological interaction networks, enabling their significant role in viral transmission dynamics [4]. Thus far, substantial efforts have focused on exploring the viral diversity of such natural reservoirs and vectors as bats, rodents, and ticks [5, 6, 7], but the understanding of viral diversity in domestic herbivores remains very limited, particularly regarding viral circulation dynamics from an ecological perspective. The knowledge gaps not only compromise our preparedness for zoonoses but also hinder livestock industry development and wildlife conservation, as they facilitate connections between humans and wildlife, enabling viral cross‐species transmission [4].

Domestic herbivores have profound biosocial linkages with humans through functions like food production, draft power, and transportation [8]. With billions of individuals occupying diverse ecological niches globally [9], they also deeply interweave with various wildlife, especially in free‐range systems [10]. These interactions provide ample opportunities for viral spillover from/to domestic herbivores, potentially impacting on human and animal health, as well as on farming communities’ livelihoods [4, 11]. Indeed, horses, camels, and cattle serve as crucial springboards for viruses like Hendra, Middle East respiratory syndrome, and influenza to jump to humans [12, 13, 14]. Pathogens can readily spread between domestic and wild herbivores through sympatric grazing and feeding [10], often leading to devastating disease outbreaks in susceptible wildlife populations [15]. For example, peste des petits ruminants virus (PPRV) spillover from sheep and goats caused thousands of endangered Mongolian saiga (*Saiga tatarica mongolica*) deaths during 2016–2020 [16]; similarly, contagious caprine pleuropneumonia caused by *Mycoplasma capricolum* subsp. *capripneumoniae* reduced the Tibetan antelope population by approximately 16% in 2012 [17]. Moreover, viral diseases remain a stubborn challenge in domestic herbivore farming. This is exemplified by the frequent emergence of mutated bovine viral diarrhea virus (BVDV) and foot‐and‐mouth disease virus (FMDV) [18, 19], introduction of exotic Akabane virus (AKAV) [20]. PPRV [21], and lumpy skin disease virus (LSDV) [22], cross‐species and cross‐regional viral spread [21], and mixed infections involving multiple viruses or even with bacteria [23]. Particularly, some notorious viruses like BVDV, PPRV, and FMDV possess broad host ranges, capable of infecting nearly all even‐toed ungulates [24, 25, 26], further complicating disease prevention and control efforts. To address the public health and food safety concerns associated with domestic herbivores, an expanded and cross‐species surveillance to uncover the true breadth and interconnectedness of viral communities circulating within herbivore populations as well as among other related species was essential.

China stands prominently as both a major player in domestic herbivore farming industries and a leading market for meat consumption [9]. with northwestern provincial regions, particularly Shaanxi, Ningxia, Gansu, Qinghai, and Inner Mongolia, being the nation's primary producing region [27]. Owing to its superior grassland coverage, historical animal farming tradition, and supportive government policies, this vast area has 35%–60% of China's cattle and small ruminant populations [27], as well as fosters more than 28 species of wild herbivores, including those protectively listed animals such as chiru, bharal, and argali [28]. To prioritize the high‐quality development of animal husbandry, China's policy frameworks have started to emphasize the integration of advanced biosecurity protocols and proactive disease management strategies in recent years. In the context, we initiated the present study with core aims to systematically characterize the viral diversity profiles of major domestic herbivore species across China's key rearing regions and to unravel the cross‐species transmission dynamics of these viruses among herbivores, humans, and wildlife, using a combination of the RNA‐specific meta‐transcriptomics (MTT) and the DNA‐specific multiple displacement amplification (MDA) viromic techniques [29]. It will provide insights into safeguarding public health and ecological civilization, as well as elevate the diagnosis, prevention, and control of viral diseases in domestic herbivores.

## 2 Results

### 2.1 Expansion of the Domestic Herbivore Virome

**FIGURE 1:**
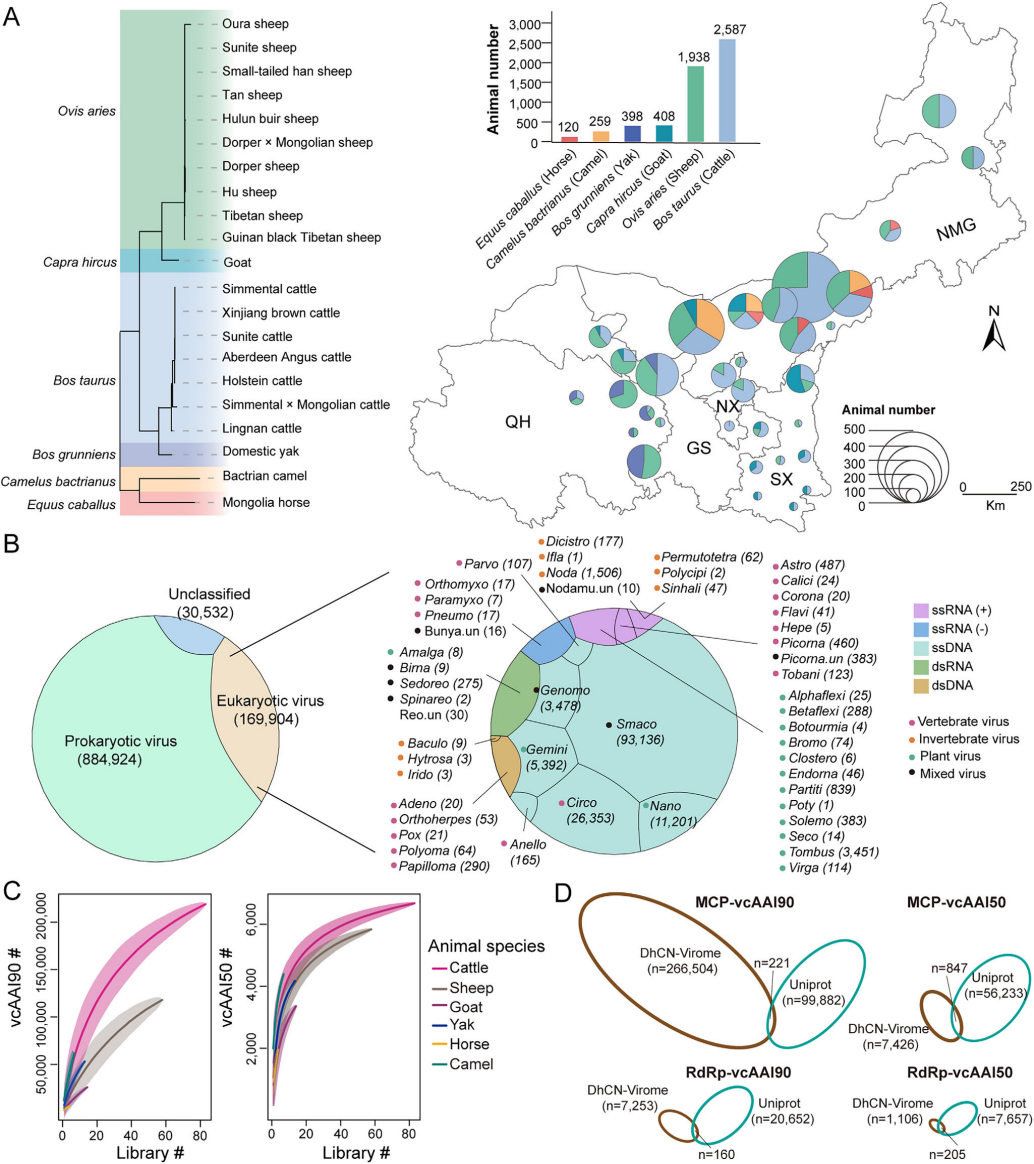
Sample information and overview of the DhCN‐Virome catalogue. (A) Phylogeny, numbers, and geographic distribution of the six domestic herbivore species. A neighbor‐joining phylogenetic tree was constructed using *Cytb* gene sequences across the analyzed breeds. Pie chart color coding corresponds to the animal number histogram. QH, Qinghai Province; GS, Gaisu Province; NX, Ningxia Hui Autonomous Region; SX, Shaanxi Province; NMG, Inner Mongolia Autonomous Region. (B) Taxonomic composition of the DhCN‐Virome catalog with total sequence counts indicated in parentheses. (C) Viral vcAAI90 (left) and vcAA50 (right) accumulation curves for the six DhSs. Shaded areas represent the 95% confidence intervals. D) Diversity expansion of DhCN‐Virome relative to the Uniprot viral branch (https://www.uniprot.org/uniprotkb).

During 2021 and 2023, a total of 5,710 adult individuals across six domestic herbivore species (DhSs), representing 10 sheep, 1 goat, 7 cattle, 1 yak, 1 camel, and 1 horse breeds, were sampled from 177 grassland farms spanning five major DhS‐producing provincial regions of China through anal, vaginal, nasopharyngeal, and/or oesophageal‐pharyngeal (OP) swab collection and blood sampling procedures (Figure 1A). We prepared 482 MTT and 482 MDA paired libraries for viromic sequencing according to animal breeds, sample types, and geographic locations. Following *de novo* assembly, multiple annotation, quality enhancement, and taxonomic assignment, we prepared the domestic herbivore viromic catalog of China (DhCN‐Virome) comprising 1,085,360 viral metagenomes (1,036,012 DNA and 49,348 RNA near‐genome‐level contigs), of which 66.7% (*n* = 735,285) were high quality. Prokaryotic viruses dominated DhCN‐Virome, accounting for 81.5% of the contigs, while eukaryotic viruses contributed 15.7% (Figure 1B). These eukaryotic viruses were very genetically diverse, with 90.8% (154,294/169,904) of contigs being annotated to 51 viral families, and the remaining 15,610 contigs represented family‐level new viruses within orders *Bunyavirales*, *Reovirales*, *Picornavirales*, etc. (Figure 1B). Owing to the proclivity of the MDA technique to amplify single‐stranded circular DNA (sscDNA) molecules [29]. Viruses with sscDNA genomes were overrepresented in DhCN‐Virome, with viral metagenomes of *Smacoviridae*, *Circoviridae*, *Nanoviridae*, *Geminiviridae*, *Genomoviridae* and *Anelloviridae* comprising 82.2% of all eukaryotic viral contigs (Figure 1B).

We identified 748,595 virus hallmark genes (VHGs) in the dataset, i.e., 13,371 RNA‐dependent RNA polymerase (RdRp) and 735,224 major capsid protein (MCP) sequences. Using the criteria of 90% and 50% average amino acid identity (AAI90 and AAI50, resembling the viral subgenus and family levels, respectively) [30], the VHGs were grouped into 274,223 AAI90 viral clusters (vcAAI90s) and 9,196 vcAAI50s. Sample size exerted a notable influence on the viromic saturation patterns across DhSs (Figure 1C). Due to their relatively small library sizes (≤ 14 libraries per species), the viral species accumulation curves of goats, yaks, camels, and horses remained unsaturated across all examined dimensions (overall, sample type, and viral cluster level) (Figure 1C; Figure S1A). In contrast, the viromic vcAAI50 compositions of cattle and sheep approached increment plateaus after sequencing 10–15 libraries at both the overall and sample type levels, except for sheep serum samples (potentially due to lower viral loads or greater individual variation) (Figures 1C; Figure S1B). It is interesting to note, however, that this pattern was not consistently observed at the vcAAI90 level (Figure S1B). DhCN‐Virome demonstrated different expansions to the global viral diversity at the subgenus and family levels, with most expansions contributed by DNA viral metagenomes (Figure 1D). Compared to UniProt, DhCN‐Virome revealed 273757 new vcAAI90s, expanding 2.3‐fold of global genetic diversity at the viral subgenus level. However, only 8532 new vcAAI50s were identified, representing a 13.1% increase in family‐level diversity relative to UniProt (Figure 1D).

### 2.2 Viromic Features Across Domestic Herbivore Species

**FIGURE 2:**
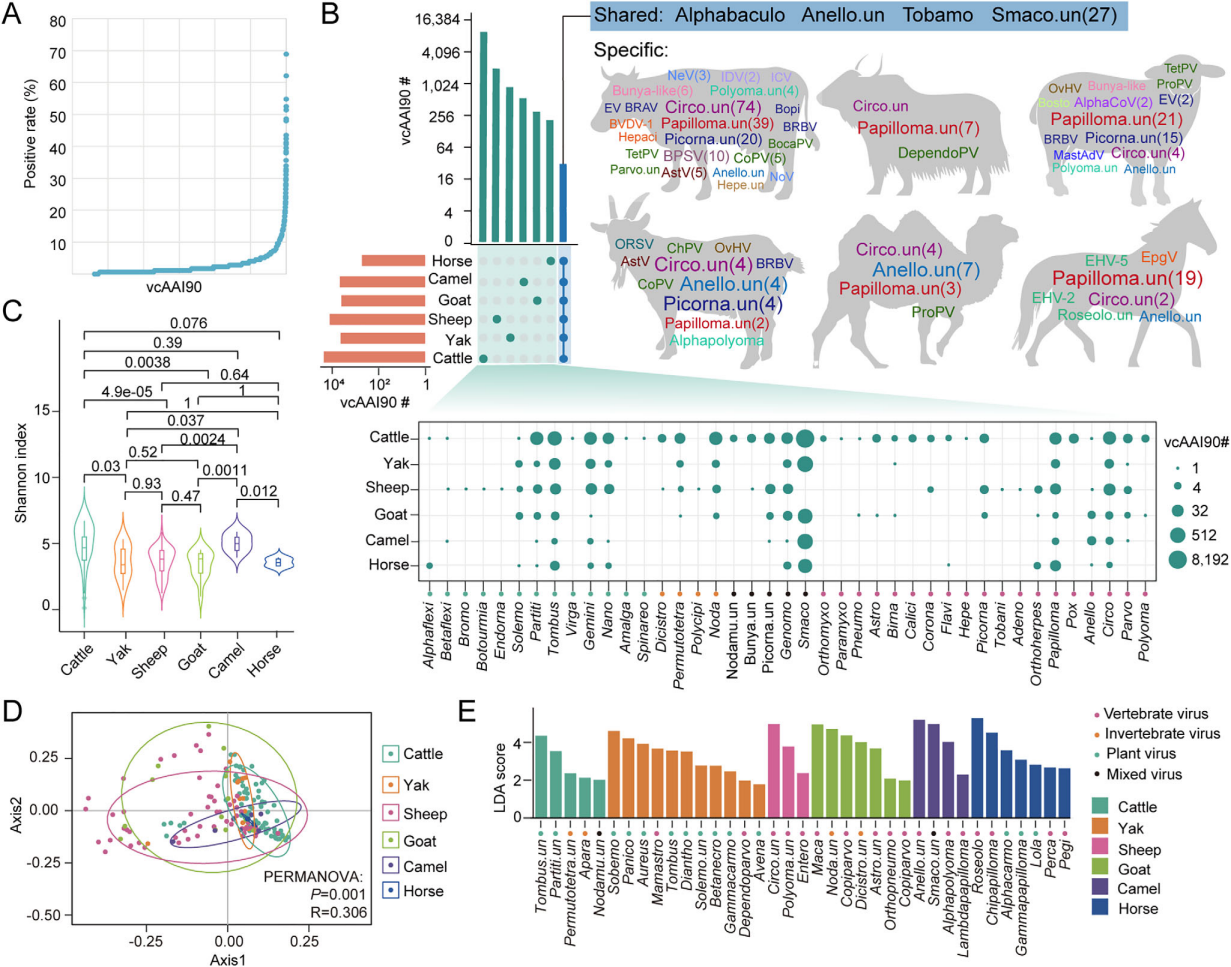
Comparison of viromic composition among the six DhSs. (A) Farm‐level positive rates of eukaryotic vcAAI90s. (B) Specific and core vcAAI90s of the six DhSs. DhS‐specific vcAAI90s (counted at viral family level) are grouped into four host‐associated groups that are differentiated using colored dots (color‐coded as in panel (E). Mammalian DhS‐specific vcAAI90s are summarized in species schematics with counts ≥ 2 indicated in parentheses. Virus name abbreviations are explained in Table S1. (C) Within‐library diversity comparison across DhSs (assessed using Shannon index), with statistical significance examined using Wilcoxon rank‐sum tests. (D) Principal coordinates analysis of vcAAI90 composition across DhSs, with group differences evaluated using PERMANOVA test (999 permutations). (E) DhS‐specific vcAAI90 signatures identified using LEfSe analysis. Color‐coded dots above vcAAI90 names denote virus groups.

Subgenus‐level viromic composition significantly varied across DhSs and sample types. DhCN‐Virome contained 26,896 eukaryotic vcAAI90s, among which 94.5% (*n* = 25,409) appeared only in ≤ 10% of all farms (Figure 2A), while just seven viral clusters related to alphabaculovirus (*n* = 1), smacovirus (*n* = 3), circovirus (*n* = 2), and tombusvirus (*n* = 1) showed widespread presence across over 50% of farms (Figure S2). Even as to each DhS, only a few eukaryotic vcAAI90s (n ≤ 196) were detected in ≥ 50% of farms (Figure S2). Among these most prevalent viruses, nearly all were identified as circular rep‐encoding single‐stranded (CRESS) DNA viruses or plant RNA viruses, with only a macavirus detected in 13/14 goat farms associated with malignant catarrhal fever and other inapparent infections in ruminants (Figure S2). We then compared the eukaryotic viromic composition across DhSs. Only 30 eukaryotic vcAAI90s were universally detected across all DhSs, with most being unclassified smacoviruses (Figure 2B). However, 13,273 viral clusters within 13 plant, 4 invertebrate, 5 host‐undetermined (designated as “Mixed viruses” in figures), and 19 vertebrate viral taxa appeared exclusively in a single DhS (Figure 2B). Plant viruses and host‐undetermined viruses constituted the overwhelming majority (96.5%, *n* = 12,807) of the DhS‐specific viruses, while vertebrate viruses represented a smaller proportion (2.4%, *n* = 315). This suggests a large portion of the virome is likely diet‐ or environment‐derived rather than representing true infecting viruses of the herbivores. The richness of DhS‐specific viruses varied significantly across the six DhSs, with cattle having the most diverse DhS‐specific eukaryotic vcAAI90s (*n* = 9,493) (Figure 2B). Probably owing to their extensive genetic diversity, circoviruses, papillomaviruses, and picornaviruses comprised the most diverse groups of DhS‐specific mammalian viruses among all the six DhSs. Notably, some DhS‐specific mammalian viruses are of health concern, e.g., bovine parainfluenza virus 3, influenza D virus, norovirus, BVDV‐1, and bovine papular stomatitis virus, which are implicated in various diseases of ruminants.

Although camels represented the second smallest group in this study (Figure 1A), they, alongside cattle, exhibited the highest eukaryotic viral richness, with 941 ± 327 (mean ± standard deviation) and 909 ± 680 eukaryotic vcAAI90s detected per farm, respectively (Figure 2C; Figure S3A). Farm scale significantly influenced viral richness in cattle but not in sheep (Figure S3B), with cattle raised in large‐scale farms harboring much richer viral communities (1310 ± 754 eukaryotic vcAAI90s per farm) compared to 648 ± 451 eukaryotic vcAAI90s per farm in smallholder settings (Figure S3B). However, neither feeding mode nor landform demonstrated significant effects on viral richness in the two DhSs (Figure S3C,D). The viral richness and within‐library diversity exhibited significant variations across different sample types, and such variations kept consistent across all studied species (Figure S3E). Anal, vaginal, and OP swabs demonstrated the highest diversity of eukaryotic viruses, with an average vcAAI90 richness ranging from 370 ± 276 to 579 ± 508 per farm across the six species. Nasopharyngeal swabs followed with ∼227 vcAAI90s per farm (Figure S3E), while serum samples exhibited substantially lower viral diversity, containing only ∼59 vcAAI90s per farm (Figure S3E).

We next compared the between‐library viromic diversity across five levels, i.e., overall, farm scale, feeding mode, landform, and sample type. The analyses revealed significant inter‐farm variation in viromic structures between small ruminant species, whereas large herbivores exhibited remarkably similar viromic composition across different farms (Figure 2D). Cattle viromes associated with the Inner Mongolia Plateau, smallholder farms, and free‐range feeding mode exhibited stronger clustering patterns, but sheep viromes were much less influenced by the landform, farm scale, and feeding mode (Figure S4). Notably, distinct compositional patterns emerged across sample types. Serum viromes displayed particularly high diversity across cattle, yaks, sheep, and camels, but notable viromic similarities were observed in anal, vaginal, and OP swabs of cattle, in anal swabs of yaks, and in vaginal swabs of sheep (Figure S4). These findings collectively indicate that domestic herbivore viromic compositions are differently influenced by body size, landform, farm scale, feeding mode, and sample type.

We then characterized viromic signatures across the five levels and found that a variety of signature viruses showed specific tropisms toward different DhSs, physiological systems or organs. Overall, yaks exhibited the highest number of DhS‐specific marker vcAAI90s (*n* = 11), with nine being plant viruses (Figure 2E). DhS‐specific markers for sheep, goats, and camels comprised nearly all vertebrate‐associated viruses, including polyomavirus, enterovirus, macavirus, and astrovirus (Figure 2E). A wide range of distinct viromic signatures emerged across sample types: anal swabs yielded the highest number of signature viruses (*n* = 34), with most being gastrointestinal viruses like torovirus, betacoronavirus, enterovirus, and hepatovirus; nasopharyngeal swabs were marked by macavirus, aphthovirus, and ten additional vertebrate/plant‐infecting viruses; serum samples displayed four characteristic viruses, such as copiparvovirus and anellovirus; and vaginal swabs were featured by five viruses, including two papillomaviruses, and one polyomavirus (Figure S5A). However, comparative analysis revealed limited marker viruses at the levels of farm scale, feeding mode, and landform, except for free‐range cattle, demonstrating 12 distinctive vcAAI90s (Figure S5B).

### 2.3 Phylogeny of DhCN‐Virome Reveals Multiple Viruses of Concern

**FIGURE 3:**
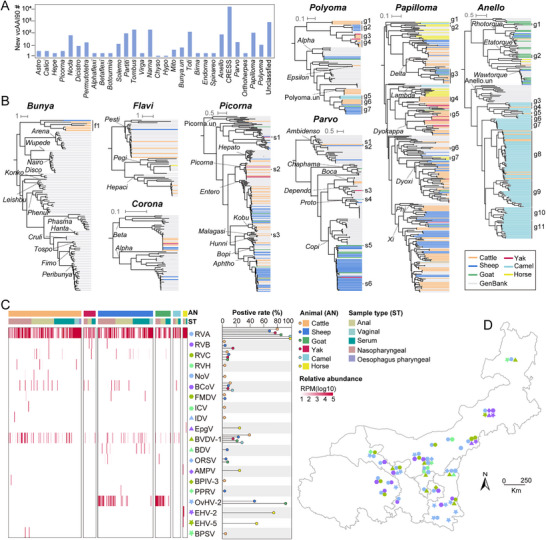
Phylogenetic analysis of DhCN‐Virome VHG sequences identifies multiple viruses of concern. (A) Candidate novel vcAAI90s detected across 30 viral taxa. (B) Maximum‐likelihood phylogenetic trees were constructed for VHG sequences from eight viral taxa. Distinct VHG clades potentially representing new species (s), genera (g), or families (f) are labeled accordingly. (C) Relative abundance patterns of 20 viruses of concern across farms and their positive rates in each DhS farms. Virus name abbreviations are explained in Table S1. (D) Spatial distribution of the 20 viruses of concern.

Comprehensive analysis of the full‐length VHG sequences showed DhCN‐Virome greatly expanded current viral diversity by identifying at least 17,430 new subgenera (Figure 3A). While mammalian viruses in domestic herbivores have been properly documented using conventional virological and metagenomic approaches [31], our investigation still revealed 219 previously uncharacterized mammalian viruses spanning at least eight families (Figure 3A). Compared to 1,299 new RNA vcAAI90s, DNA viral diversity showed dramatic expansion (*n* = 15,753) (Figure S6A). Particularly, viruses with a circular genome contributed at least 15,734 new subgenera. We examined the phylogenetic relationships of VHG sequences within 19 family or order groups (Figure 3B; Figure S6B). Most DhCN‐Virome contigs within families *Flaviviridae*, *Coronaviridae*, *Astroviridae*, *Tobaniviridae*, *Paramyxoviridae*, *Sedoreoviridae*, *Pneumoviridae*, *Poxviridae*, *Adenoviridae*, and *Orthoherpesviridae* exhibited high similarities (> 85% average nucleotide identity (ANI)) with currently circulating strains of veterinary significance. Notably, some betacoronaviruses from cattle, yaks, and sheep; mamastroviruses from the six DhSs; and rotaviruses group A (RVAs) from sheep, goats, and cattle each showed up to 99% ANI across hosts, suggesting their possibility of cross‐species transmission. In contrast, lots of contigs from families *Polyomaviridae*, *Parvoviridae*, *Anelloviridae*, and *Papillomaviridae* formed distinct clusters positioned between established taxonomic groups, indicating potential novel species or genera within these families. Notably, a large anellovirus cluster comprising predominantly camel‐associated contigs displayed substantial divergence from known viruses with an AAI of < 35.0% (Figure 3B). In addition, a cluster of bunya‐like contigs and a diversity of CRESS DNA molecules did not fall into any known families, indicating family‐level member additions to their respective orders. The bunya‐like cluster consisted of six cattle‐derived and one sheep‐derived contigs and was phylogenetically placed within the class *Bunyaviricetes*, showing AAIs of ≤ 12.7% with known bunyaviruses (Figure 3C; Figure S6C). They preserved characteristic RdRp motifs of *Bunyaviricetes* members (Figure S6D), justifying their family‐level addition(s) within the class. Molecular detection revealed broad circulation of the novel bunyaviruses in annual swabs of ten cattle populations in Inner Mongolia, Gansu, Ningxia, and Shaanxi, with detection rates ranging 6.7%–66.7%.

The phylogenetic analyses demonstrated that many DhCN‐Virome contigs were closely related to viruses known or suspected to cause human and animal diseases. Further identification revealed 20 viruses within DhCN‐Virome responsible for diarrhea in both human infants and young livestock (RVA), bovine respiratory infections (influenza C/D viruses), and highly lethal and contagious diseases of small ruminants (FMDV and PPRV), etc. (Figure 3C). The distribution patterns of the viruses of health concern varied substantially across farms and animal species (Figure 3C,D). Diarrhea‐related RVA, rotavirus group B, rotavirus group C, bovine coronavirus (BCoV), and BVDV were detected in at least three DhSs, suggesting their potential cross‐species transmission. Especially, RVA and BVDV exhibited broad dissemination across farms, except for the absence of BVDV in horses. Most of these pathogens (70.0%, 14/20) demonstrated relatively low prevalence rates within their respective host species (Figure 3C). However, RVA, equid pegivirus (EpgV), avian metapneumovirus (AMPV), ovine herpesvirus 2 (OvHV‐2), and equid herpesviruses 2 and 5 (EHV‐2, EHV‐5) showed notably higher prevalence, being detected in over 20% of farms within their corresponding host populations (Figure 3C,D). Notably, we identified bovine papular stomatitis virus (BPSV), a pathogen of the *Parapoxvirus* genus causing bovine papular stomatitis and previously undocumented in China [32], in two geographically distinct farms in Ningxia. The virus showed 95.8%–99.9% ANIs with German BPSV strains, suggesting a possible exotic introduction event. In addition, metapneumoviruses, causing respiratory tract infections in humans and birds, have not been documented in other animal hosts [33]. The AMPV‐like virus detected in horses showed 81.1%–88.2% ANIs with mallard strains, suggesting a potential avian‐to‐horse transmission route [34].

### 2.4 Virus Circulation Among Domestic Herbivores and Other Taxa

**FIGURE 4:**
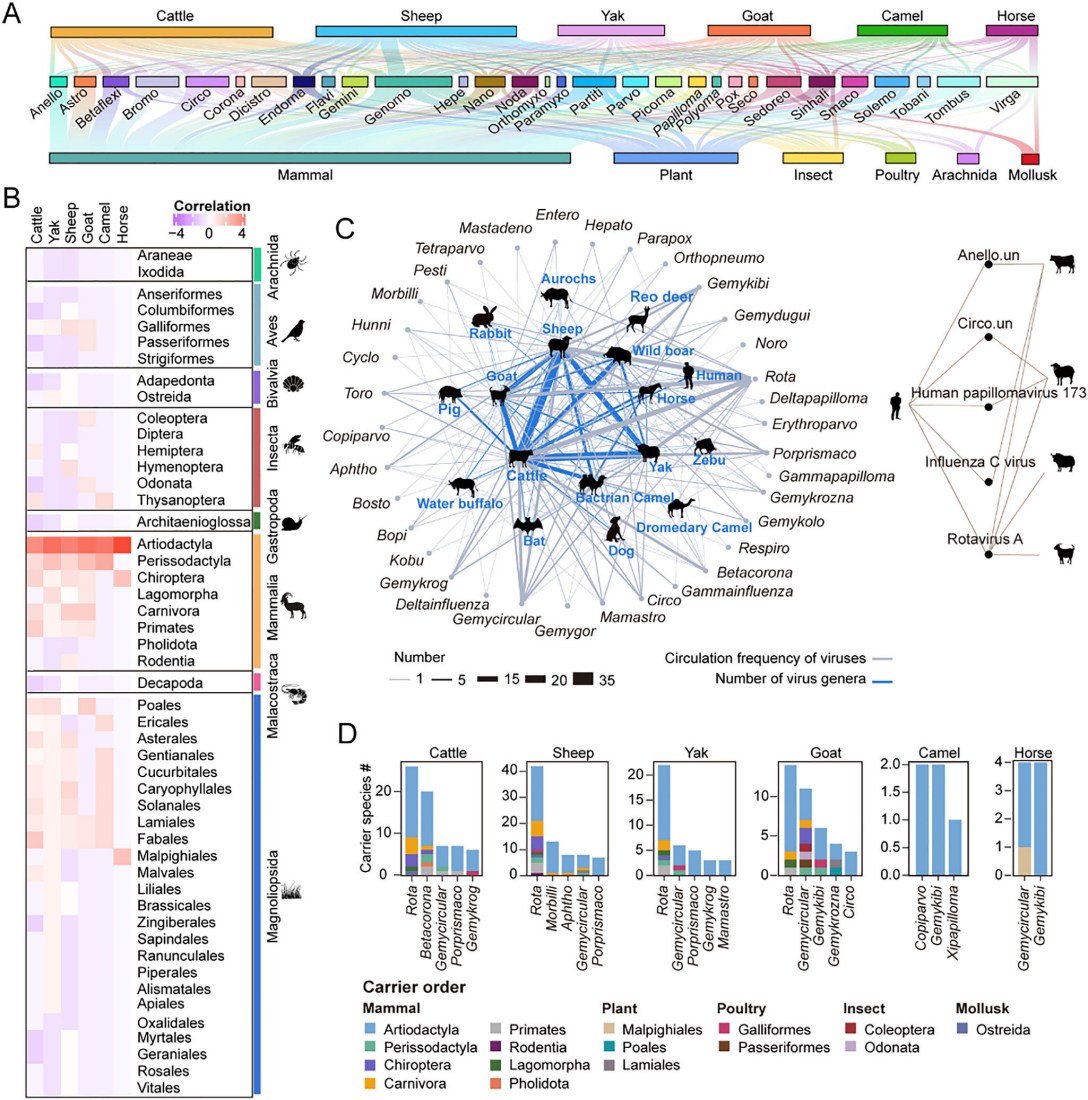
Circulation dynamics of DhCN‐Virome. (A) Viruses from 30 families across six DhSs exhibit associations with carriers spanning six taxonomic groups. (B) Correlation analysis of viral circulation networks demonstrates that viruses in the six DhSs are primarily associated with animals of the order Artiodactyla. (C) Circulation networks of mammalian viruses, collapsed at the genus level, identify five human‐animal transmitting viruses. (D) Carrier species diversity among the top five mammalian viruses (collapsed at the genus level) with the broadest host ranges across six DhSs. Camel and horses only detected 3 and 2 circulating mammalian viruses, respectively.

The cross‐species transmission of some health‐concerning viruses prompted us to thoroughly investigate the global circulation range and dynamics of DhCN‐Virome‐associated viruses across all eukaryotic organisms. Generally, we identified 67,873 DhCN‐Virome contigs covering 37 viral families related to at least 350 species of mammals, plants, insects, birds, arachnids, and mollusks (referred to as “carriers”) [35] (Figure 4A). These viruses showed various relatedness patterns. Anelloviruses, astroviruses, coronaviruses, geminiviruses, hepeviruses, orthomyxoviruses, paramyxoviruses, papillomaviruses, polyomaviruses, poxviruses, smacoviruses, and tobaniviruses were exclusively associated with mammals, while viruses from other families displayed multi‐taxa connections across the six carrier categories (Figure 4A). Correlation analysis showed that viruses of the six DhSs were mostly related to artiodactyls, followed by perissodactyls, carnivores, chiropterans, primates, and lagomorphs within the class Mammalia, then to ten plant orders within the class Magnoliopsida (Figure 4B). This indicates intensively internal circulation of viruses among herbivores.

Further genus‐level analysis mapped the circulation of 35 vertebrate viral genera across 17 mammalian species (Figure 4C). In the viral circulation networks, the most diverse viral circulation occurred among cattle, sheep, and yaks, covering 20–35 viral genera, followed by cattle‐wild boar, goat‐sheep, cattle‐camel, and sheep‐wild boar pairs, with 15–18 viral genera. Notably, among the 20 viruses‐of‐health‐concern identified, while EpgV, EHV‐2, and EHV‐5 showed exclusive association with horses, the remaining 17 were related to multiple animal species, including such wild herbivores as wild boars, reo deer, aurochs, etc. For example, BVDV from the genus *Pestivirus* circulates among pigs, cattle, goats, sheep, and wild boar populations; FMDV (type O) from the genus *Aphthovirus* transmits among diverse even‐toed ungulates; and noteworthy is betacoronavirus, which was primarily related to cattle yet also documented in nine additional species. Human‐related viral links were identified in cattle, sheep, yaks, and goats through 6, 2, 1, and 1 viral genus, respectively (Figure 4C). Notably, contigs of anellovirus, circovirus, papillomavirus, influenza C virus, and rotavirus in cattle, sheep, yaks, and goats showed 90.5%–98.0% ANIs with human viruses, markedly higher than the < 90% ANIs with their herbivore‐associated neighbors, suggesting potential cross‐species transmission of the viruses between humans and these domestic herbivores (Figure 4C).

The circulating viruses showed a distinct carrier spectrum. Plant‐infecting cucumoviruses, tobamoviruses and alfamoviruses were related to a wide range of plants spanning 25–80 species (Figure S7A). Among vertebrate‐associated viruses, rotaviruses of cattle, sheep, yaks, and goats demonstrated the most extensive circulation range, infecting 14–42 species across orders Artiodactyla, Chiroptera, Carnivora, and Primates (Figure 4D). Betacoronaviruses of cattle and morbilliviruses of sheep circulated in 20 and 13 mammalian species, respectively (Figure 4D). Notably, nearly all vertebrate‐associated viruses from the six DhSs were mainly related to other members within the order Artiodactyla (Figure 4D), suggesting that cross‐species viral transmission events are more frequent among these evolutionarily closely related species.

### 2.5 Diverse Function of Prokaryotic Viruses in DhCN‐Virome

**FIGURE 5:**
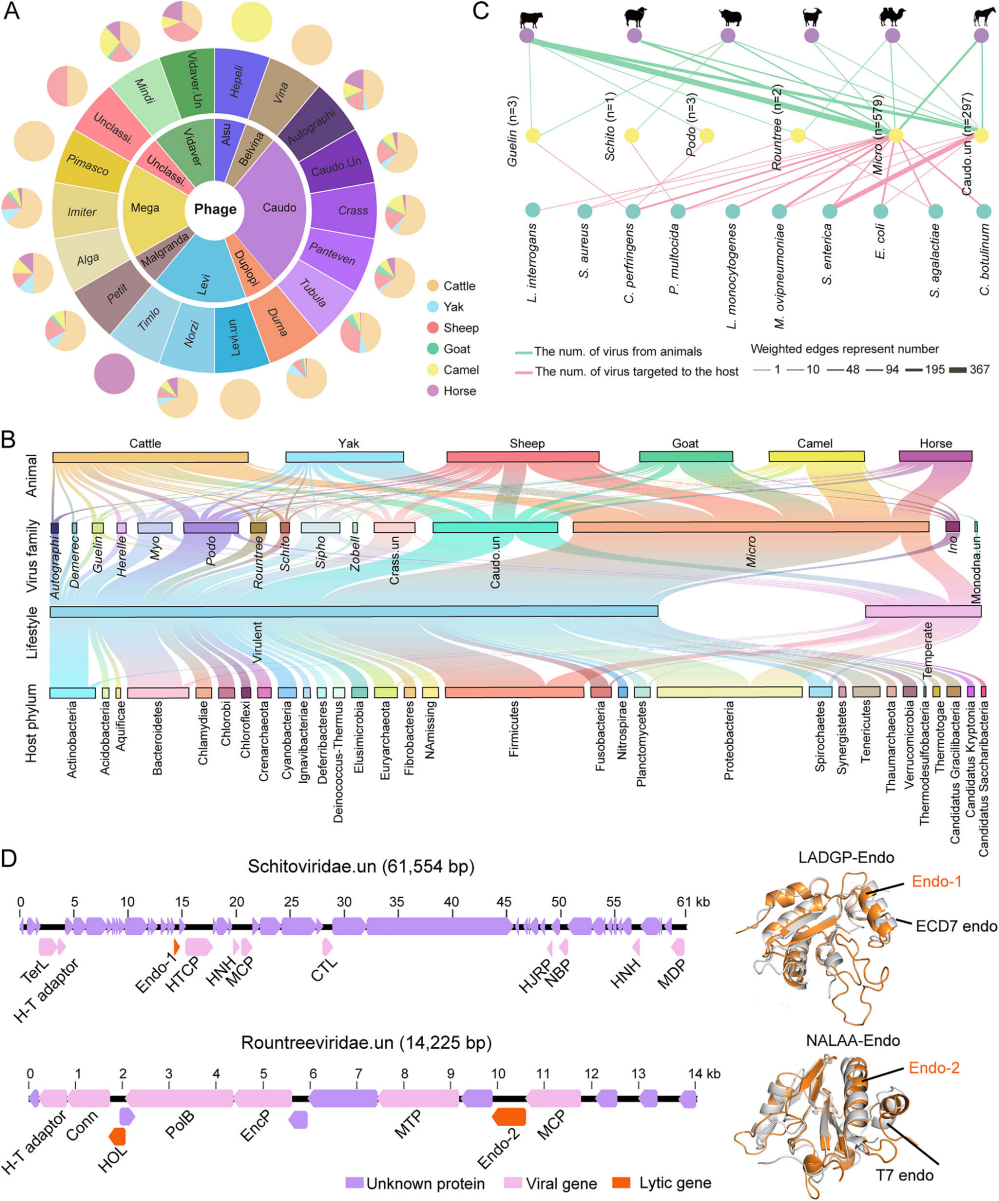
Identification and characterization of DhCN‐Virome bacteriophages. (A) Taxonomic distribution of bacteriophages detected in the six DhSs. (B) Lifestyle characteristics and putative host associations of bacteriophages across six DhSs. (C) Lytic bacteriophages demonstrating host specificity toward pathogenic bacteria. (D) Genomic frameworks of two novel bacteriophages exhibiting minimal sequence homology to known viruses, with structural analysis of their predicted lytic enzymes.

Bacteriophages are the predominant biological entities in DhCN‐Virome with a remarkable diversity (*n* = 884,924) (Figure 1B). The bacteriophages were taxonomically classified into at least 38 families across 14 orders within eight classes encompassing all genome types, with cattle exhibiting higher phage diversity (Figure 5A). Taxonomic characterization at finer resolution remained challenging, with only 0.6% (5,541/817,237) assignable to established genera (Figure S8). The majority (75.0%) of the bacteriophages showed no detectable host associations through current bioinformatic approaches, the remaining 25.0% matched up with at least 28 bacteria phyla, predominantly targeting Proteobacteria, Firmicutes, Bacteroidetes, and Actinobacteria (Figure 5B). Most characterized host‐phage interactions involved virulent (lytic) lifecycle strategies (Figure 5B), suggesting these viruses primarily function as bacterial predators. Further examination of these hosts identified several clinically relevant herbivore pathogens, such as *Clostridium perfringens*, causing gas gangrene of various animals, and *Mycoplasma ovipneumoniae* causing ovine pneumonia [36]. Microviruses (*n* = 579) and unclassified caudoviruses (*n* = 297) constituted the predominant phage groups attacking the bacterial pathogens (Figure 5C). Structural and functional characterization of phage‐encoded lytic genes revealed two distinct lytic protein family (Holin and Endolysin) exhibiting 27.0%–100% AAIs to reference lytic factors. Despite sequence divergence in some cases, conserved structural architectures were maintained at critical functional domains through homology modeling (Figure 5D), particularly within catalytic sites essential for host cell lysis, justifying the predicted bacteriolytic capacity of the phage‐encoded enzymes.

## 3 Discussion

Disease control and prevention serve as a cornerstone for the healthy development of animal husbandry. This study contributes the most fundamental knowledge to decode the sector's most concerning issues: the background of risk viruses hidden in apparently healthy livestock and their circulation dynamics. All these are bases for conceiving and implementing precision measures to control and prevent viral infectious diseases of livestock. Using our combined viromic protocol, DhCN‐Virome substantially expands our understanding of herbivore adult‐associated viral diversity. Unlike the expansion of the bat viromic catalogue BtCN‐Virome by 3.4 and 7.6 folds at subgenus and family levels, respectively [35], DhCN‐Virome expanded by only 13.6% the family‐level diversity, compared to a 2.3‐fold expansion at the subgenus‐level (Figure 1D). The contrasting diversity patterns between the subgenus and family levels suggest that herbivore viral diversity expands primarily at lower taxonomic ranks within the existing viral family framework, which also explained that the viral diversity of cattle and sheep reached saturation at the family level after sequencing 10–15 farms but continued increasing at the subgenus level as the number of farms grew (Figure 1C). This may stem partially from the inherent capacity of herbivore viruses to explore and expand sequence space during frequent cross‐species transmission between different ruminants and in response to host vaccination [37, 38].

To predict viral disease risks, we identified 20 health‐concerning viruses, including high‐consequence FMDV, PPRV, BVDV, etc., and deciphered their geographic distribution (Figure 3C,D). These pathogenic viruses are primarily associated with diarrhea, while also being linked to reproductive failure, respiratory infection, and mucosal lesions, etc. These disease associations are largely shaped by our cross‐sectional sampling strategy, which focused on mucosal swabs and serum samples that favor the detection of actively replicating, shed viruses. Consequently, latent viruses like bovine herpesvirus 1 were likely underrepresented in our viromic profiling. This highlights that multiple types (e.g., tissue specimens) of longitudinal sampling are necessary for systematic disease surveillance campaigns. Co‐infections with the viruses were frequently observed with some viruses capable of infecting multiple DhSs (Figure 3D). Due to extensive vaccination against FMDV and PPRV, both viruses were detected only on a limited number of farms without causing disease outbreaks, indicating effective vaccine protection. However, it is important to note that cryptic infections with these viruses can challenge vaccine effectiveness, increasing the risk of immune escape and potential disease resurgence. Notably, we detected the exotic BPSV, a parapoxvirus not previously documented in China. This virus is associated with papular and erosive lesions on the muzzle, lips, and oral mucosa of cattle, posing a dual threat to production performance and operational costs [39]. The virus can also infect humans occupationally, causing skin lesions [32], highlighting the need to systematically survey the virus for both veterinary and public health benefits. However, beyond BPSV and RVA, we detected no other viruses overtly pathogenic to humans. This suggests that domestic herbivores pose minimal zoonotic viral threats to humans, likely due to long‐term viral co‐adaptation over thousands of years of coexistence [8, 40]. However, given the extensive diversity of novel viruses identified and the key role of domestic herbivores in viral transmission, it is imperative to sustain ongoing surveillance at the human‐domestic herbivore interface. These findings reflect the complicated situation of viral diseases in China's domestic herbivore breeding. To mitigate these challenges, regular surveillance and an appropriate vaccination strategy should be strengthened.

Viral cross‐species transmission is a critical driver of emerging/re‐emerging infectious disease outbreaks [41]. Analysis of viral sharing networks revealed extensive connectivity between these herbivore species and diverse taxa (Figure 4A), with dietary factors, particularly plant consumption, contributing to certain ecological links [35]. While we cannot rule out the possibility of shared environmental exposure as an alternative to direct host‐to‐host transmission, the mammalian virus‐mediated connections strongly support the cross‐species transmissibility of these viruses. Notably, the five human‐related viruses in these herbivore species demonstrated anthroponotic transmission of viruses from humans to livestock during farming practices [42], underscoring that humans function not merely as recipients but also as active participants in viral host‐switching dynamics [43]. Viral transmission primarily occurred among herbivore species (Figure 4B,C), and indeed, these herbivore species establish a stable ecosystem due to shared dietary habits and frequent close contact, particularly in free‐range farming systems [44]. Hence, viruses readily disseminate among these evolutionarily related species. Although most circulating viruses show no direct disease association, notable exceptions include such viruses of concern as BVDV, PPRV, FMDV, etc., which exhibit not only widespread prevalence across the DhSs but also persistent circulation within wild ruminant populations (Figure 4C). These findings emphasize that partitioning these DhSs into single species is unfeasible during domestic herbivore disease management. Given the current agricultural mode and ecological context, all such DhSs must be treated collectively, with implications extended to wildlife conservation efforts [45, 46]. Therefore, a multispecies, sector‐wide strategy is essential to effectively manage herbivore epizootics, including integrated surveillance networks, coordinated vaccination programs across species, and tailored cross‐sectoral biosecurity protocols for China's mixed‐farming systems.

The stability of microbial communities exhibits a complex dependence on environmental openness, wherein moderately open ecosystems can maintain stable microbiota under specific conditions [47, 48]. Our study revealed a “higher openness, greater stability” pattern in domestic herbivore viromes, evidenced by remarkable virome similarity across cattle populations in different farming practices, i.e., confined versus free‐range feeding mode (Figure S4A). This pattern consistently extended across sample types, particularly anal swabs versus serum. Anal swabs, representing open host‐environment interfaces, demonstrated highly convergent viral communities, whereas serum samples from enclosed internal environments showed significantly greater virome diversity (Figure S4B). This phenomenon is likely attributed to open environments facilitating viral transmission between animals and their surroundings, while enclosed environments impose stronger selective pressures that shape distinct viromic profiles [49, 50]. Notably, large herbivores (e.g., cattle and yaks) displayed greater virome similarity compared to small ruminants (Figure 2D). This distinction may be ascribed to that small ruminants are often maintained at higher population densities. Dense populations may enhance resistance to external microbial perturbations, thereby having more characteristic viromic signatures [51]. These findings hold practical implications for implementing precision livestock management. As intensive industrialized production popularizes, with livestock increasingly raised in high‐density confined systems, animals tend to harbor more farm‐specific viromes that are highly susceptible to environmental influences. Consequently, effective disease prevention and control in modern animal husbandry necessitates enhanced biosecurity protocols and refined microbial management strategies.

Bacteriophages, as predominant components of viral communities, have received limited attention in animal disease control and prevention [31]. Although not directly impacting animal health, these entities influence host physiology through microbiome modulation [52]. The global rise of antimicrobial resistance, largely driven by veterinary antibiotic overuse, has made “antibiotic reduction and replacement” a critical objective in modern animal husbandry [53]. Phage‐based therapies have reemerged as viable solutions for bacterial infections, particularly due to their natural abundance in microbial ecosystems [54, 55]. Like the compositional dominance of virulent phages in human gut phageomes [56, 57], our analysis of DhCN‐Virome revealed that lytic phages constitute the predominant viral population (Figure 5B). These phages represent a critical resource for identifying novel therapeutic agents against antibiotic‐resistant bacteria. Notably, we identified multiple phage candidates with specific lytic activity against clinically relevant bacterial species (Figure 5C), suggesting their potential utility as precision biotherapeutics for managing bacterial infections in veterinary contexts.

## 4 Conclusion

This study decoded the viral baseline of major domestic herbivores via integrated RNA and DNA viromic techniques, advancing our understanding of viral diversity harbored by clinically healthy herbivore populations. Characterization of pathogenic viruses provides direct guidance for formulating personalized immunization and disease prevention strategies in livestock farms. Particularly, our findings reveal intensive viral circulation among herbivores, highlighting the need for holistic disease control in domestic herbivores and wildlife conservation. Additionally, the abundant bacteriophage resources generated in this study expand the toolkit for bacterial disease control in agricultural settings. This work bridges fundamental virome ecology and applied livestock management, laying a foundation for proactive One Health‐based strategies to mitigate cross‐species transmission risks and safeguard herbivore health.

## 5 Experimental Section

### 5.1 Ethics Statement

The procedures for sampling were reviewed and approved by the Administrative Committee on Animal Welfare of the Changchun Veterinary Research Institute (Institutional Animal Care and Use Committee Authorization, permit number: AMMS‐11‐2021‐068).

### 5.2 Sample Collection

Sample collection methodology closely followed our established protocols for previous pig‐based research [58]. Sampling efforts encompassed 120 horses, 259 Bactrian camels, 398 yaks, 408 goats, 1,938 sheep, and 2,587 cattle from 177 farms across five major domestic herbivore‐producing regions: Inner Mongolia Autonomous Region (number of farms = 76), Ningxia Hui Autonomous Region (*n* = 20), Gansu Province (*n* = 33), Qinghai Province (*n* = 25), and Shaanxi Province (*n* = 23). These vast regions cover three landform types, i.e., Inner Mongolia Plateau (1000–1200 m a.s.l.), Loess Plateau (1000–2000 m a.s.l.), and Qinghai‐Tibet Plateau (3200–3700 m a.s.l.). Farm selection criteria required: (i) ≥ 3 years of operational history in domestic herbivore production; (ii) maintenance of an annual livestock inventory (ALI) exceeding 50 animals; and (iii) no clinical disease outbreaks reported during the last 12 months. Farm scale classification referred to the *Scale Standards and Filing Management Measures for Livestock and Poultry Farms* proposed by the Ministry of Agriculture and Rural Affairs of China [59]. Large‐scale operations were defined as having an ALI of ≥ 100 large herbivores (cattle, camels, yaks, and horses) or ≥ 300 small ruminants (goats and sheep); remaining facilities were considered smallholder farms. Participating farm owners or on‐site veterinarians signed confidentiality agreements to ensure data anonymity. Field documentation included comprehensive records of farm characteristics and vaccination protocols. From each farm, specimens comprising paired anal, vaginal, nasopharyngeal, and/or OP swabs supplemented with 2 mL serum samples were systematically collected from 30–40 adult individuals. As a result, a total of 4,410 anal, 4,410 nasopharyngeal, 1,005 vaginal, and 400 OP swabs and 4,304 blood samples were collected. All specimens underwent cryogenic preservation during transport, with subsequent storage at ‐80°C until laboratory analysis. Animal breeds were morphologically identified and further validated by sequencing the cytochrome b (*Cytb*) gene [60], with resultant data utilized for phylogenetic reconstruction.

### 5.3 Sample Preprocessing and Viromic Sequencing

Our prior evaluations indicated that combining DNA‐specific MDA and RNA‐specific MTT viromic approaches provided enhanced viromic profile coverage while maintaining ecologically meaningful conclusions [29]. Consequently, all specimens were subjected to paired MDA and MTT viromic library preparation. Each swab specimen was shaken with 2 mL DMEM buffer at 4°C overnight to facilitate viral particle release. Equal volumes of swab eluates and corresponding serum samples from each farm were pooled to achieve a final volume of 2 mL per sample type. Resulting mixtures were centrifugated at 10,000×g for 10 min at 4°C, followed by filtration through 0.45 µm pore membranes (Millipore) to remove cellular debris. To preserve viral nucleic acid (NA) for successful library preparation, we omitted the free NA digestion step, which often leads to library preparation failure with swab and serum samples. Instead, filtrates were directly partitioned into two aliquots, one for MDA processing and the other for MTT analysis. To inspect cross‐contamination risks, a Vero cell control was included for every 40 pools and processed identically. For MDA, DNA was extracted using the DNeasy Blood & Tissue kit (Qiagen, Düsseldorf, Germany) with subsequent isothermal amplification via the Illustra GenomiPhi V2 DNA amplification kit (GE, Fairfield, CT). To mitigate amplification bias toward cssDNA, reactions were incubated at 30°C for 1.5 h followed by thermal inactivation at 65°C for 10 min. Purified products (1 µg) were subjected to library preparation using the NEBNext Ultra DNA Library Prep Kit. For MTT, total RNA extraction utilized TRIzol reagent (TaKaRa, Dalian, China) with magnetic bead‐based purification. Ribosomal RNA was depleted using the Ribo–Zero Magnetic Gold Kit (Epicentre Biotechnologies, Madison, WI), followed by RNA quantification via Qubit 4 fluorometer (Invitrogen, Carlsbad, CA). Meta‐transcriptomic libraries were constructed using the NEBNext Ultra Directional RNA Library Prep Kit (NEB, Ipswich, MA). All libraries incorporated 10 nt unique dual indexes to minimize index hopping during multiplex sequencing, with paired‐end 150‐bp sequencing performed on NovaSeq 6000 (Illumina, San Diego, CA) or DNBSEQ‐T7 (BGI, Shenzhen, China) platforms.

### 5.4 Data Pretreatment and de Novo Assembly

We generated 3.3 tera bases (Tb) MTT and 6.3 Tb MDA high‐quality data for viromic analyses. All raw FASTQ files were filtered using FastQC v.0.20.0 to remove adapters and low‐quality reads. MTT data were subsequently subjected to host genome assignment and microbiome classification before proceeding to *de novo* assembly. Reference assemblies for cattle (GenBank accession: GCF_002263795.3), yak (GCA_005887515.3), sheep (GCA_019145175.1 and GCA_017524585.1), goat (GCA_015443085.1), camel (GCF_000767855.1), horse (GCF_002863925.1), and African green monkey (GCA_023783515.1) were sourced from the NCBI Genome database. Each reference genome was independently indexed to construct Bowtie2‐compatible databases. High‐quality reads from individual MTT libraries were aligned to corresponding reference databases using Bowtie2 v.2.4.1 in “–very‐sensitive‐global” alignment mode. Unassigned reads were subsequently subjected to rapid taxonomic classification for bacterial, archaeal, and fungal taxa via Kraken2 v.2.0.9‐β with the RefSeq database (March 2022 release). MDA‐generated data were excluded from host and microbial genome separation to maintain potential viral‐host genome integration events. Both remaining MTT reads and MDA datasets underwent iterative assembly. For each farm, multi‐sample reads were grouped according to sequencing library type and assembled using MEGAHIT v.1.2.9 under default parameters. To enhance contig continuity, a secondary assembly round was performed with Geneious Prime v.2022.2.2 using customized parameters (≥ 99% identity over 30 bp overlaps). For circular DNA recovery, MDA‐derived contigs were processed through SCAPP v.0.1.4 without scoring or gene annotation features. Assembly quality was evaluated using QUAST v.5.2.0 across all datasets. Final sequences (≥ 500 and 1500 bp for MTT and MDA contigs, respectively) from individual farms were dereplicated using MMseq2 v.e2840992948ee89dcc336522dc98a74fe0adf00 with ≥ 99% similarity over 80% coverage relative to the shorter sequence.

### 5.5 Virus‐Like Sequence Recovery

The annotation methodology for MTT contigs followed the protocol established during BtCN‐Virome preparation [35]. Briefly, MTT contigs were translated into amino acid (aa) sequences using Prodigal v.2.6.3 with the standard genetic code table and meta mode. Resulting open reading frames (ORFs) ≥ 50 aa in length were queried against our eukaryotic viral reference database (EVRD v.3.0) via DIAMOND BLASTP v.2.1.9. Sequences yielding significant BLASTP hits (e‐value ≤1 × 10^−^^5^) were provisionally classified as virus‐like sequences (VLSs). Additionally, all aa sequences were analyzed using hmmscan (HMMER v.3.3) against curated HMM profiles integrating RdRp models from iVirus, the virus branch of eggNOG v.5.0, and RdRp‐scan. Sequences achieving bit scores ≥ 30 against these models were also considered VLSs. MDA contigs underwent parallel processing: aa translation was performed for all sequences, followed by DIAMOND BLASTP analysis against the virus‐branch of UniProt. Sequences with significant matches (e‐value ≤ 1 × 10^−^^5^) were tentatively classified as VLSs. To detect evolutionarily divergent viral signals, all aa sequences were subjected to two machine learning‐based analyses: convolutional neural network‐based DeepVirFinder v.0.0.2 (default parameters) and deep neural network‐based geNomad v.1.7.6 (end‐to‐end mode). Sequences meeting either DeepVirFinder criteria (score ≥ 0.9 and *p*‐value ≤ 0.01) or geNomad thresholds (score ≥ 0.9, ≥ 1 viral gene, or ≥ 2 viral hallmark genes) were considered VLSs.

### 5.6 Quality Control

To ensure dataset quality, we implemented multiple quality control measures targeting false‐positive annotations, cross‐library contamination, and exogenous contaminants originating from environmental sources or reagents. During sample preprocessing, we incorporated 12 MTT and 12 MDA Vero cell blank controls that underwent parallel sequencing and bioinformatic analysis. Comparative analysis of clinical sample group (CSG) VLSs against the control group counterparts was performed using BLASTn and BLASTp algorithms. No known domestic herbivore‐specific viruses were detected in control group VLSs, indicating minimal risk of cross‐contamination during preprocessing or index hopping in multiplexed high throughput sequencing workflows. However, we did find hundreds of thousands of hits between the two VLS sets associated with unclassified *Riboviria* spp., uncultured viruses, *Ortervirales* spp., and environmental viruses, etc., with 71.8%–100% similarities, which were probably false positives (though a few likely being bona fide viruses) and subsequently excluded from CSG datasets. Second, we performed competitive annotation of CSG VLSs using BLASTx against a curated reference database containing EVRD, genome assemblies of the six herbivore species, human reference genome GRCh38.p14 (accession no.: GCF_000001405.40), and RefSeq viral/fungal/archaeal/bacterial branches. For each local region, we retained the top 25 high‐scoring pairs (HSPs) and classified regions as viral if > 50% of HSPs mapped to viral references. VLSs were categorized as viral only if ≥ 90% of their annotated constituent regions met this criterion. Finally, during EVRD database construction, we prepared a collection of problematic viral sequences derived from vector backbones/functional cassettes, reagent‐associated viral sequences, and host genomic contaminants. All remaining CSG VLSs were subjected to BLASTN screening against this collection, with sequences exhibiting ≥ 99% identity across ≥ 400 nt alignments to any entry in the collection being excluded. We then evaluated the remaining VLSs using Checkv v.1.0.3, which detected 6670 host contamination sequences. This multi‐layered filtration protocol ensured robust removal of false positive annotation while preserving authentic viral sequences. These sequences were dereplicated using MMseqs2 at 95% nt similarity over 80% coverage, yielding the final viral contigs (*n* = 1085360) designated DhCN‐Virome. The DhCN‐Virome sequences had an average length of 4,892 bp (range: 500–71835), with 19%–100% amino acid (aa) identity with known references in UniProt (Figure S9). Of these viral contigs, geNomad identified the largest proportion (66.3%, *n* = 718700), followed by BLAST (31.8%, *n* = 345006), while HMMER and DeepVirFinder contributed 8676 and 3978 VLSs, respectively. Viral contigs in DhCN‐Virome were considered high‐quality if they possessed nearly complete genomes (i.e., exhibiting genomic structures similar to relatives that are complete genomes, particularly those with ≥ 10 nt inverted terminal repeats or circular conformation) or were classified as high‐quality by Checkv.

### 5.7 Taxonomical Assignment

We employed a hierarchical strategy to assign taxonomic lineages to DhCN‐Virome viral contigs [35]. VHG sequences within DhCN‐Virome were identified as described elsewhere [35, 61]. Taxonomic assignment was first performed based on VHGs. All DhCN‐Virome VHGs were clustered with reference sequences from EVRD and UniProt's viral branch using MMseqs2 at the AAI90 and AAI50 levels. Clustered DhCN‐Virome contigs were assigned genus or family names derived from reference sequences within their vcAAI90 or vcAAI50 clusters. The remaining DhCN‐Virome contigs were then queried against EVRD and UniProt's viral branch using DIAMOND BLASTx in ultra‐sensitive mode. The lowest common ancestor taxon was determined based on the top five blast hits using Taxonkit v.0.8.0, which was subsequently assigned to the query sequence. Finally, viral sequences recovered through machine‐learning analysis were taxonomically classified using geNomad Tax information.

### 5.8 Abundance‐ and Richness‐Based Ecological Analyses

All abundance‐ and richness‐based analyses utilized the VHG‐containing DhCN‐Virome contigs. Host genome assignment and microbiome classification of MTT and MDA high‐quality reads were performed as described in the “Data pretreatment and de novo assembly” section. Unclassified reads from each dataset were mapped against the VHG‐containing DhCN‐Virome contigs using Bowtie2 with the end‐to‐end sensitive preset. Mapped reads were counted using SAMtools v.1.10. The relative abundance (RPM) of each sequence was derived by dividing the number of million unclassified reads by its read count; values ≤ 1 were set to 0 to further minimize index hopping. All VHG sequences were then clustered at AAI90 and AAI50 levels using MMseqs2. Subgenus‐level and family‐level relative abundances were obtained by summing the RPM values of all sequences within the vcAAI90 and vcAAI50 clusters, respectively. To generate farm‐level viromic overviews, the maximum RPM value for each vcAAI90 or vcAAI50 cluster across all sample types within a farm was selected using Pandas v.2.2.3, representing the cluster's relative abundance in that farm. Saturation statuses of viral clusters were evaluated at all levels using the *specaccum* function (vegan v.1.12 package) in random mode with 1000 permutations. The viral cluster increment was calculated by subtracting the viral cluster count of n libraries from that of n + 1 libraries using the *diff* function. Increments were visualized using ggplot2 and curve‐fitted using ggtrendline v.1.0.3.

We used the vcAAI90‐level RPM table to evaluate alpha and beta diversities, core virome composition, and viromic signatures across all investigated levels. Within‐library diversity metrics (i.e., viral cluster richness, Shannon–Wiener index, and Gini–Simpson index) were calculated for each library using the *diversity* function. Between‐group and overall differences of these metrics were assessed using Wilcoxon rank‐sum tests and Kruskal–Wallis tests, respectively. Between‐library diversity was evaluated by computing Bray–Curtis distance matrices of RPM tables using the *vegdist* function, followed by principal coordinates analysis (PCoA) with the *cmdscale* function. Factor contributions to sample differences were quantified using the *adonis* function with the PERMANOVA test under 999 permutations. Viromic composition across six species was compared through Upset analysis (UpSetR v.1.4.0). Viromic signatures were extracted using LEfSe analysis implemented in microeco v.1.10.0 and magrittr v.2.0.3.

### 5.9 Phylogenetic Analysis

All phylogenies were inferred using full‐length VHG sequences at the viral family level, except for bunyaviruses and CRESS DNA viruses analyzed at class and phylum levels, respectively. DhCN‐Virome VHG representatives for all taxa were sampled at vcAAI90, except for CRESS DNA viruses sampled at vcAAI50 due to high redundancy at vcAAI90. Representatives were queried against our EVRD database using BLASTp to identify closest relatives. Within each taxon, DhCN‐Virome VHG representatives and their relatives were aligned using MAFFT v.7.520 (E‐INS‐i strategy) and trimmed using trimAL v.1.4 to remove columns containing ≥ 50% gaps. Maximum‐likelihood trees were then inferred with IQ‐TREE v.1.6.12 using 1000 ultrafast bootstrap replicates and automatically selected best‐fit models via ModelFinder. We employed two approaches to ensure phylogenetic robustness. Initial trees were manually inspected to verify the grouping of ICTV‐approved genus representatives and detect unrelated taxa. In addition, alignments were examined in Geneious Prime v.2022.2.2 (Clustal Omega v.1.2.2) to confirm key motifs across sequences. Sequences from unrelated taxa or lacking conserved motifs were removed, with the remaining sequences subjected to another round of phylogenetic reconstruction.

### 5.10 Identification of Viruses of Concern

We defined viruses evidently or potentially associated with human or animal diseases as viruses of concern. DhCN‐Virome contigs were queried against the virus branch (ViPR) of the Bacterial and Viral Bioinformatic Resource Center (BV‐BRC) (v.3.30.19a) [62] using BLASTn. Sequences with species‐level similarity (≥ 90% ANI) to reference viruses were retained and further screened against China Catalogue of Animal Pathogenic Microorganisms [36]. Farms containing the sequences with RPM >1 were considered positive for viruses of concern.

### 5.11 Carrier Determination and Circulation Networks

We previously prepared a virus‐carrier (resembling virus‐host) relationship database by compiling host metadata from gbvrl source annotations of GenBank and the Virus‐Host DB repository [35]. This resource was augmented with host association data derived from our established bat (BtCN‐Virome) [35] and wild boar (BrCN‐Virome) [61] virome datasets. DhCN‐Virome contigs were queried against this database using BLASTn. All species‐level hits (i.e., ≥ 90% similarity across ≥ 50% query coverage) were retained. Query sequences were then assigned carrier information from all matching BLASTn subjects. To visualize the circulation networks, we collapsed these circulating viruses at the genus level and prepared network files based on the top ten mammal species carrying the most vertebrate‐associated virus genera linked to the six domestic herbivores. Within the networks, connections between the six domestic herbivores and other mammals, and between circulating virus genera and their carriers, were represented as edges. Edge weights reflected the number of virus genera circulating between carriers and the viruses’ circulation frequency. These files were processed in Gephi v.1.10.1 and visualized using the Fruchterman‐Reingold layout.

### 5.12 Analyses of Bacteriophages

Bacteriophage analyses were conducted at the farm population level by combining all sample types collected from each farm to capture the integrated bacteriophage profile of the entire herbivore population within a farm. To analyze bacteriophage diversity in DhCN‐Virome at the genus level, we merged the prokaryotic virome with greater than 50% completeness (*n* = 817,237) according to the Checkv results with the prokaryotic viral RefSeq database (release 94) and performed an all‐versus‐all DIAMOND search. This was followed by protein clustering using the Markov clustering algorithm (MCL) and virus clustering using ClusterONE v2 within the vConTACT2 pipeline [63]. The resulting network was visualized in Gephi. Bacterial hosts were predicted using the CHERRY module in PhaBOX v.2.1.10 with a score cutoff ≥ 0.5. Pathogenic bacteria were identified based on China's Catalogue of Animal Pathogenic Microorganisms. Bacteriophage lifestyles were predicted using multiple approaches. It was initially predicted with BACPHLIP v.0.9.6 and the PhaTYP module in PhaBOX. Discrepant predictions between methods were excluded. Putative lytic bacteriophages were validated by identifying lytic genes using geNomad, with hits further confirmed by BLASTp search against UniProt. Lytic bacteriophages targeting pathogenic bacteria were subjected to structural comparison of lytic genes against the RCSB Protein Data Bank (PDB). Structures were modeled using the Swiss‐Model server and visualized in PyMOL v.3.1.4.1.

### 5.13 Molecular Detection and Validation

Molecular detection was used to determine individual positive rates or validate viromic results for specific viruses. Primer pairs were designed in Geneious Prime based on contigs (Table S2). Total NAs were extracted using the RaPure Viral RNA/DNA Kit. Reverse transcription was conducted using the PrimeScript cDNA Synthesis Kit (TaKaRa) following the manufacturer's protocols. PCR reactions used 2 × Rapid PCR Master Mix (Tiangen) with thermal cycling: 95°C for 5 min; 30 cycles (outer PCR) or 35 cycles (inner PCR) of 95°C for 15 s, 56°C (or adjusted per primer pairs) for 15 s, and 72°C for 15 s; final extension at 72°C for 5 min. Distilled water served as a negative control; positive controls were not included. Target amplicons were subcloned into pMD18‐T vectors (TaKaRa) and transformed into DH5α competent *E. coli* cells (Tiangen). Five clones per amplicon were randomly selected for bidirectional Sanger sequencing on an ABI 3730xl Genetic Analyzer (ComateBio).

### 5.14 Statistical Analysis

All ecological and statistical analyses were performed using R v.4.1.3. Groups with insufficient sample sizes were excluded in statistical comparisons to ensure robustness. Statistical testing included the Wilcoxon test, Kruskal–Wallis test, and PERMANOVA test for two or multiple comparisons. Differences between groups were considered statistically significant at *p* < 0.05.

## Author Contributions

B.H. and C.T. conceived and designed the study. Yong Li, B.T., Yuanqing Lin, L. Y., G.Z., J.L., Y.G., L.L., J.C., W.T., L.J., M.F., G.M., F.D., X.Z., C.T., and B.H. collected samples. Y.S., Yuhang Liu, and Z.S. performed the experiments. Y.S. and B.H. analyzed the data. B.H., Y.S., and C.T. drafted the paper. B.H. and C.T. revised the paper.

## Funding

National Natural Science Foundation of China (32130104), Qinghai Science and Technology Achievement Transformation Special Project (2025‐NK‐112).

## Conflicts of Interest

The authors declare no conflicts of interest.

## Supporting information




**Supporting File**: advs74989‐sup‐0001‐SuppMat.docx.

## Data Availability

All raw data generated by Illumina sequencing have been deposited in the Science Data Bank at https://doi.org/10.57760/sciencedb.25407. Representative sequences of DhCN‐Virome and amplicon sequences have been deposited in the Science Data Bank at https://doi.org/10.57760/sciencedb.28232. The full spectrum of DhCN‐Virome and phylogenetic files are available on Science Data Bank at https://doi.org/10.57760/sciencedb.28580.
