# Supplementary material for: Diversity Patterns of Domestic Herbivore Viruses in China Reveal Transmission Dynamics with Disease Management Implications
Source: Adv Sci (Weinh). 2026 Mar 25;13(32):e17444. doi: 10.1002/advs.202517444 (PMC13252600; doi:10.1002/advs.202517444)
Supplement: Supplementary file 1 — Supporting File: advs74989‐sup‐0001‐SuppMat.docx. [file ADVS-13-e17444-s001.docx]

Supporting Information

**Diversity patterns of domestic herbivore viruses in China reveal transmission dynamics with disease management implications**

*Yue Sun, Yong Li, Bagen Temur, Yuanqing Lin, Yuhang Liu, Le Yi, Zheng Sun, Gang Zhang, Jun Li, Yu Guo, Linchuan Li, Jinshan Cai, Wenliang Tian, Gen Meng, Lingling Jiang, Min Fang, Fuying Ding, Xuezhang Zhou*^*^*, Changchun Tu*^*^*, Biao He*^*^


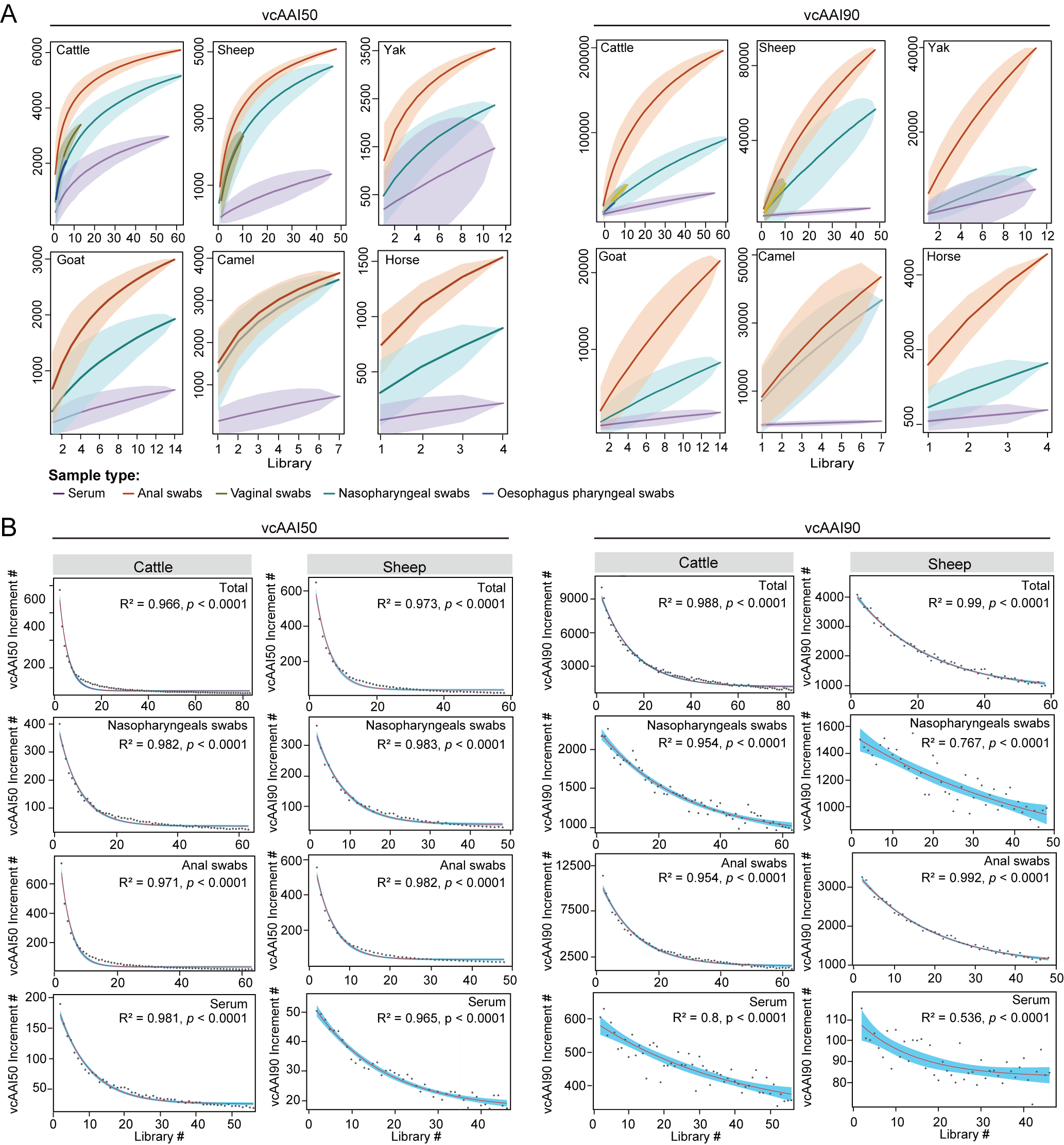


**Figure S1.** Viromic saturation assessment of different samples across the six DhSs. A) vcAAI50 and vcAAI90 level saturation assessments. Colored lines indicate different sample types. Shaded areas represent the 95% confidence intervals. B) vcAAI50 and vcAA90 increment trends with expanding sequencing library size. Exponential curves are used for optimal fitting with shaded areas indicating the 95% confidence intervals.


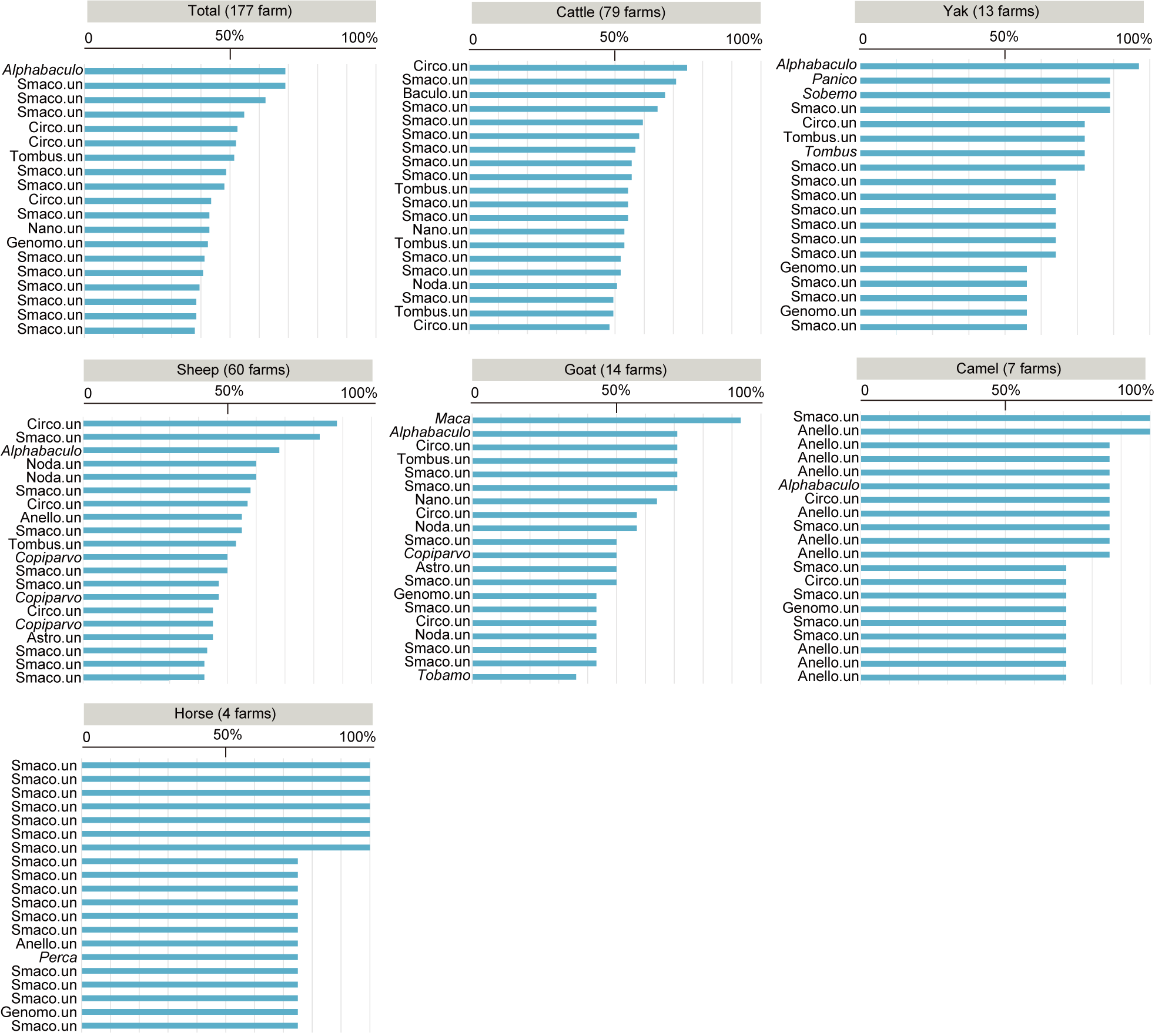


**Figure S2.** The top 20 vcAAI90s with the highest positive rates across total and individual DhS farms.


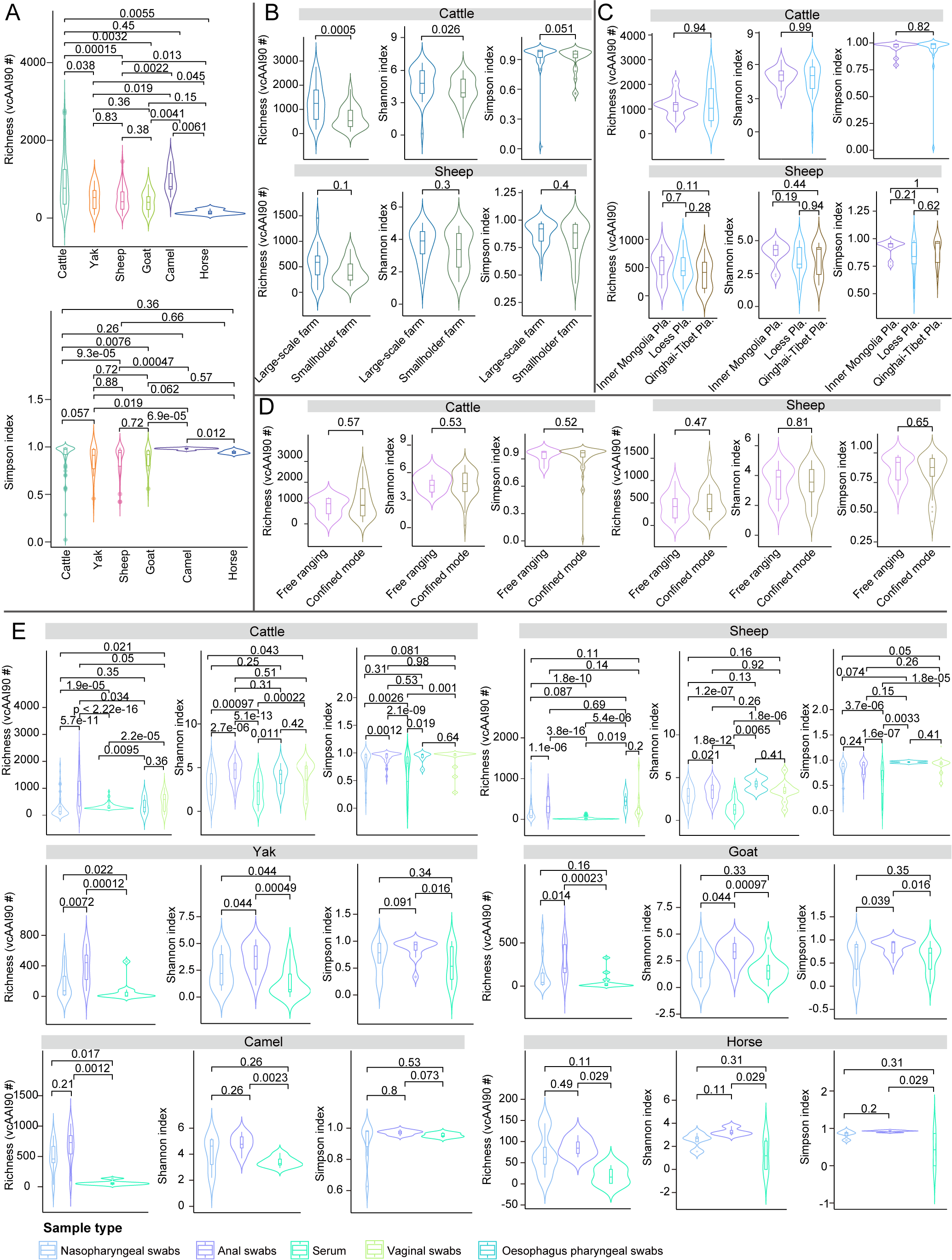


**Figure S3.** Comparison of vcAAI90-level alpha diversities using species richness, Shannon index, and Simpson index. A) Comparison of alpha diversity (richness and Simpson index) across the six DhSs. B) Comparison of alpha diversity between large-scale farms and smallholder farms. C) Comparison of alpha diversity between different landforms. D) Comparison of alpha diversity between free ranging mode and confined mode. E) Comparison of alpha diversity between different sample types. Statistic differences were examined using Wilcoxon rank-sum tests.


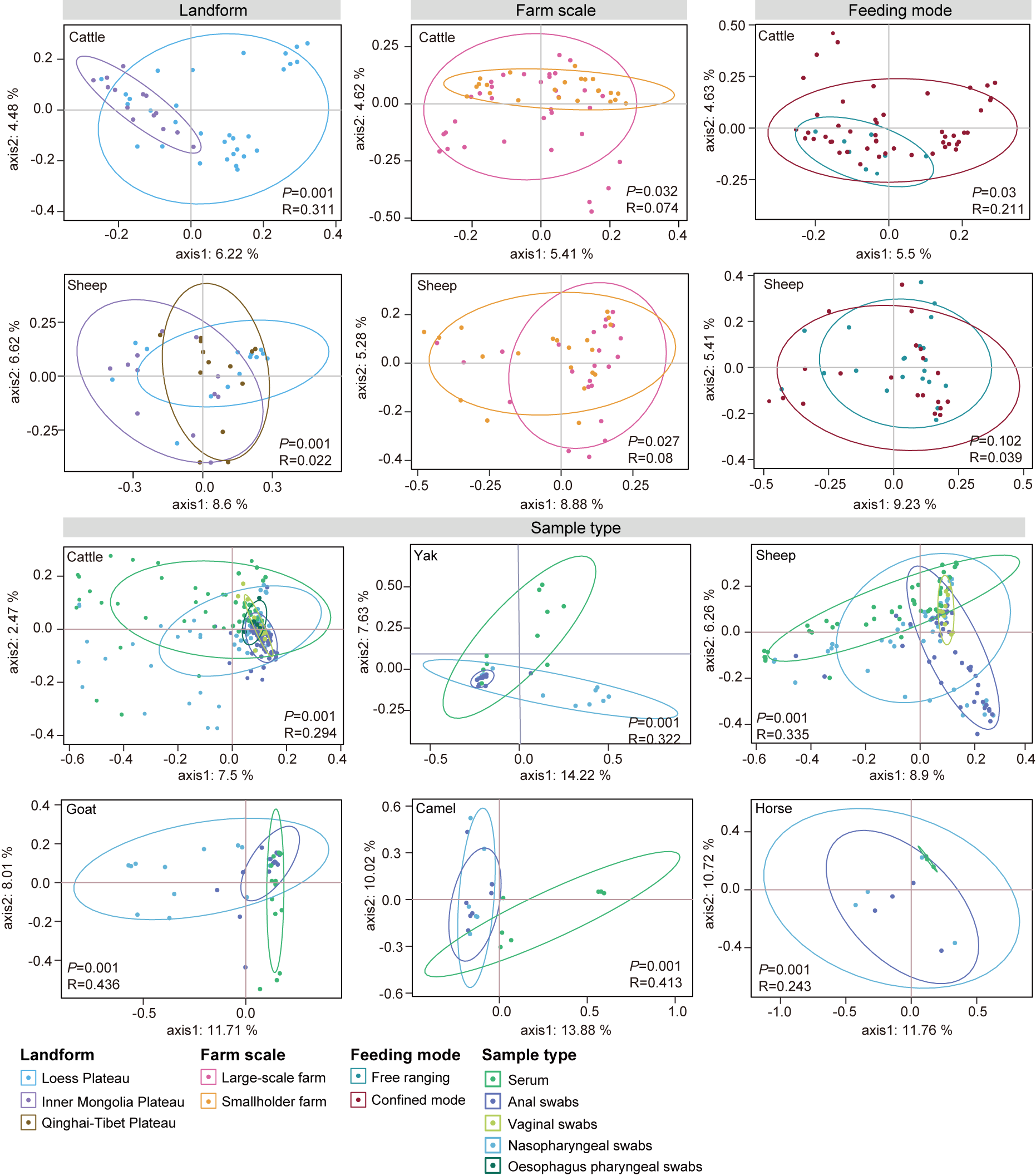


**Figure S4.** Principal coordinates analyses (PCoA) of vcAAI90 composition across landforms, farm scales, feeding modes, and sample types, with group differences evaluated using PERMANOVA test (999 permutations). Color schemes for these dots are shown at the bottom of the figure.


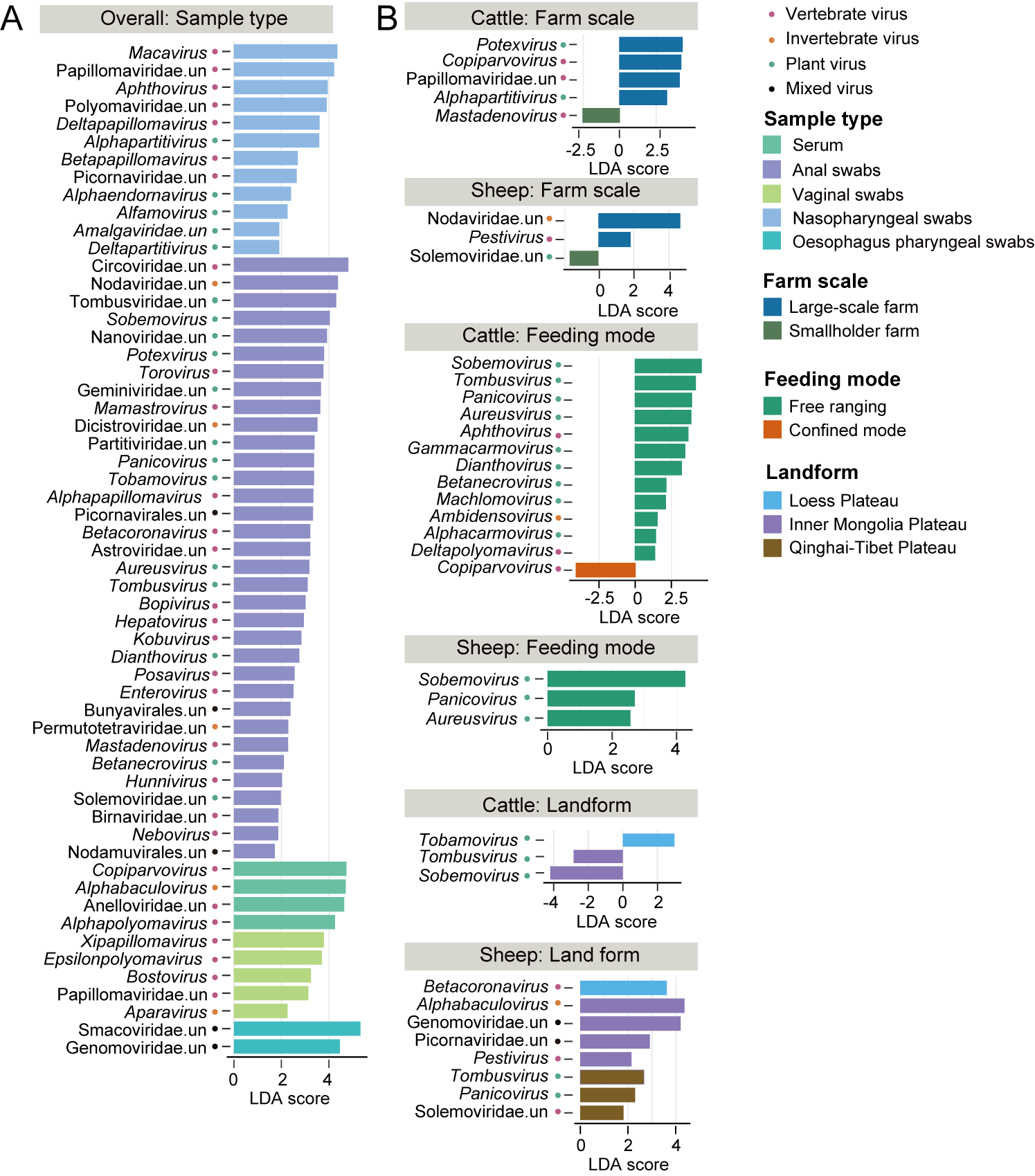


**Figure S5.** Viromic signatures identified using LEfSe analyses across different levels. A) Sample type-specific vcAAI90 signatures across the six DhSs. B) Farm scale-, feeding mode-, and landform-specific vcAAI90 signatures in cattle and/or sheep. Colored dots behind vcAAI90 names indicate host-associated viral groups. Color schemes are shown on the right of the figure.


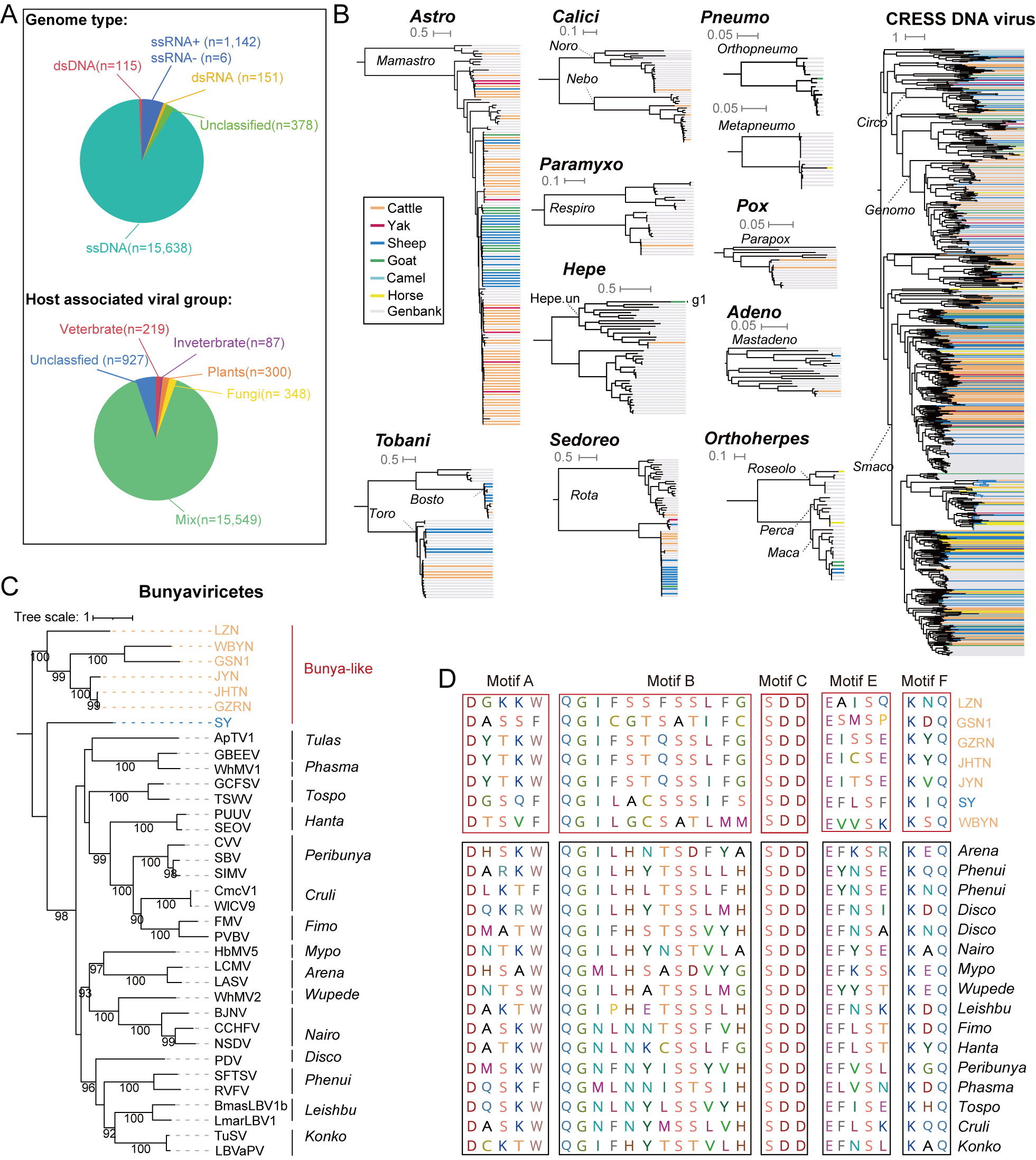


**Figure S6.** Diversity and phylogeny of DhCN-Virome VHG sequences. A) Numbers of new eukaryotic vcAAI90s at the genome type and host-associated viral group levels. B) Maximum likelihood phylogenetic trees of VHG sequences from 11 viral taxa. C) Detailed phylogenetic analysis of bunya-like VHG sequences. Colored tips indicate animal species from which the viruses were detected (color scheme shown in panel B). Virus name abbreviations are explained in supplementary table 1. D) Sequence comparison of key RdRp motifs of new bunya-like VHG sequences (colored names; color scheme shown in panel B) with their counterparts of known members within the phylum *Bunyavirales*.

**
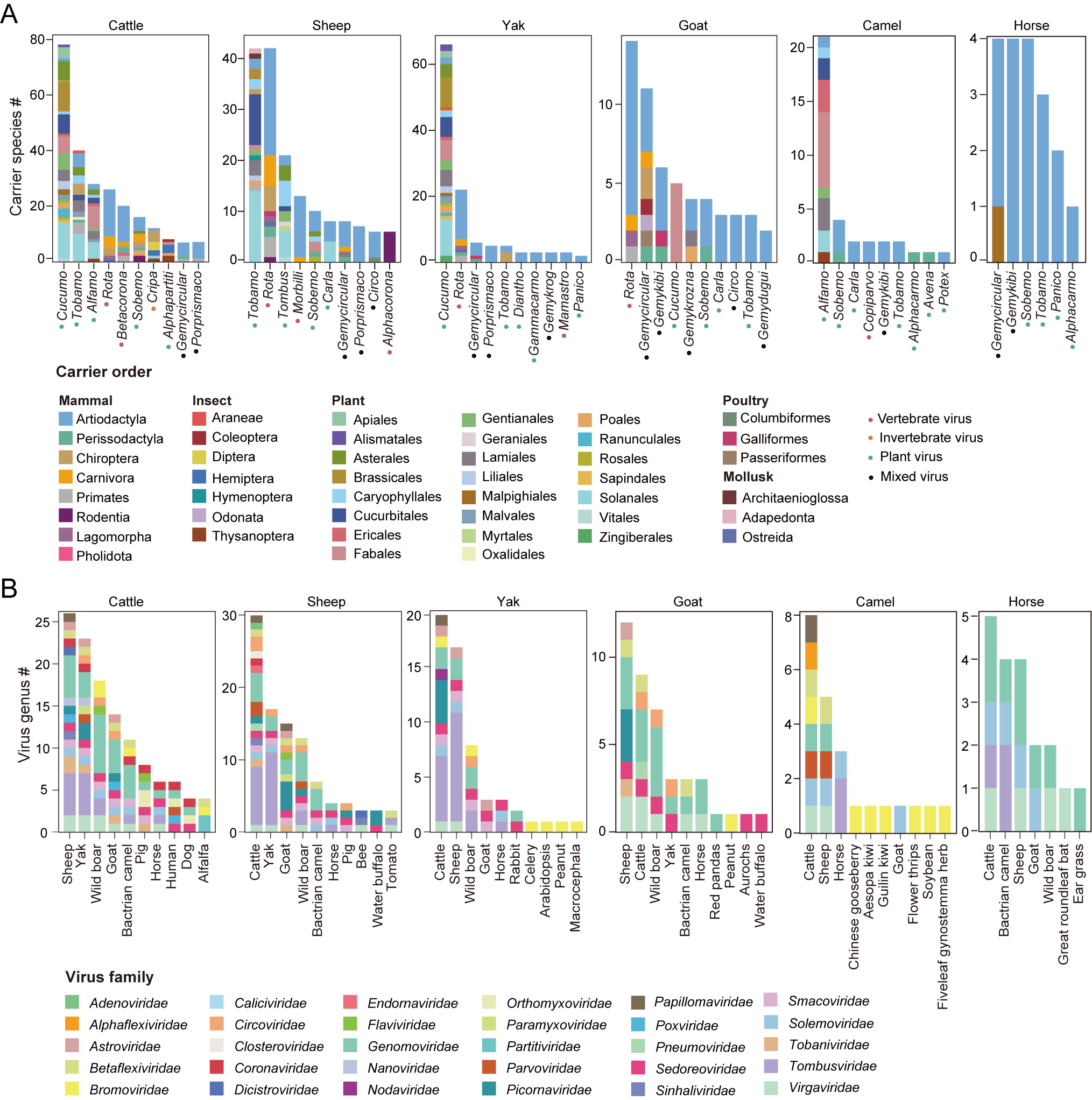
**

**Figure S7.** Carrier species diversity of viruses and viral diversity circulating between animals. A) Carrier species diversity of the top 10 eukaryotic viruses (collapsed at the genus level) with the broadest host ranges across the six DhSs. Horses were identified with only 6 eukaryotic viral genera. B) Eukaryotic viral diversity of the top 10 species with the most viruses circulating with the six DhSs. Horses were identified with only 7 viral-interacting carrier species.


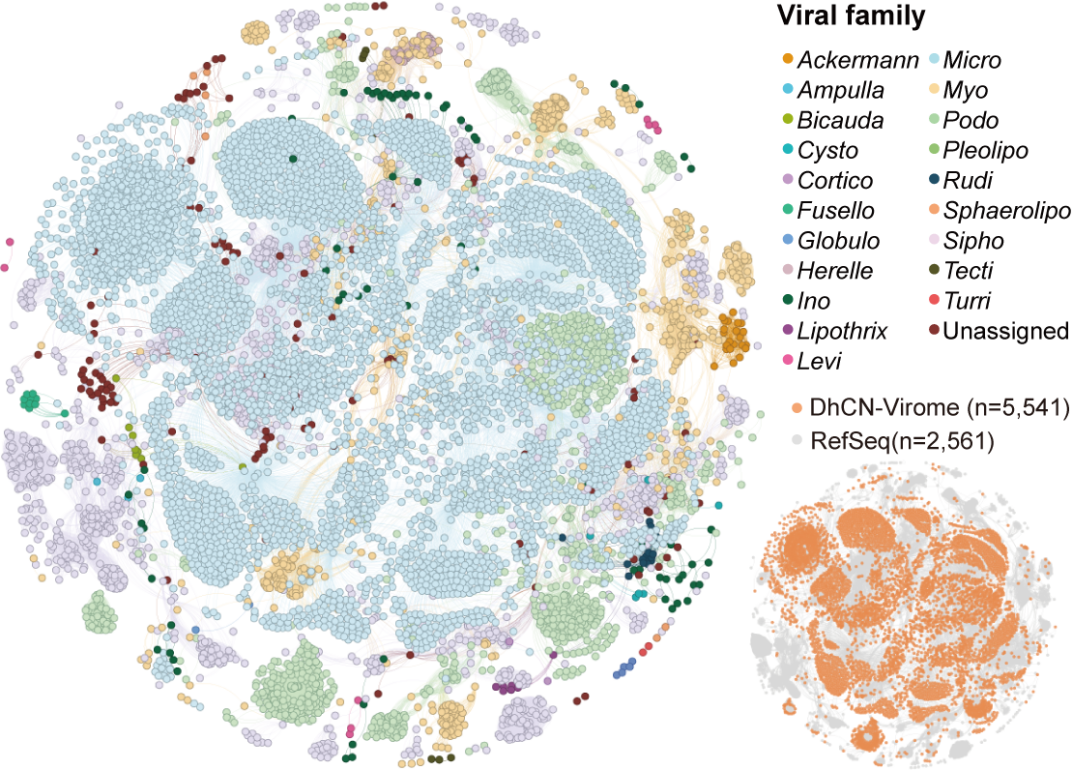


**Figure S8.** Viral genus-level classification of DhCN-Virome bacteriophages.


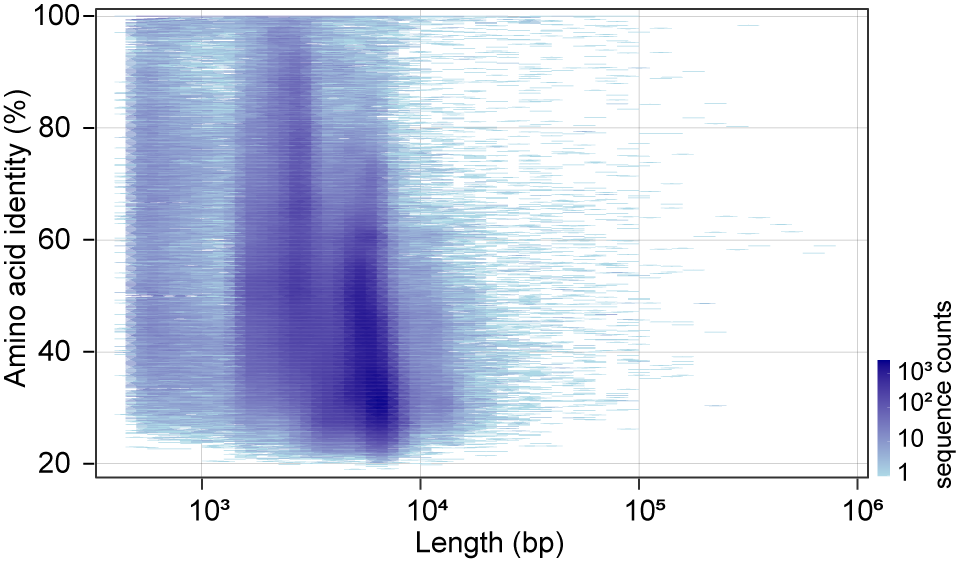


**Figure S9.** Length and identity distributions of DhCN-Virome sequences.

**Table S1. Abbreviations used in this study.**

| Abbreviation | Explanation | Abbreviation | Explanation |
| --- | --- | --- | --- |
| LADGP | L alanyl D glutamate peptidase | LASV | Lassa virus |
| AlphaCoV | Alphacoronavirus | LBVaPV | Lactuca big vein associated phlebovirus |
| NALAA | N acetylmuramoyl L alanine amidase | LCMV | Lymphocytic choriomeningitis virus |
| ApTV1 | Agrocybe praecox tulasvirus 1 | LmarLBV1 | LmarLBV1 |
| AstV | Astrovirus | MastAdV | Mastadenovirus |
| BDV | Border disease virus | MCP | Major capsid protein |
| BJNV | Běijí nairovirus | MDP | Predicted metal-dependent peptidase |
| BmasLBV1b | Blechomonas maslovi leishbunyavirus 1 | MTP | Major tail protein |
| BocaPV | Bocaparvovirus | NBP | Phage nucleotide-binding protein |
| BPIV-3 | Bovine parainfluenza virus 3 | NeV | Nebovirus |
| BRAV | Bovine rhinitis A virus | NoV | Norovirus |
| BRBV | Bovine rhinitis B virus | NSDV | Nairobi sheep disease virus |
| BVDV-1 | Bovine viral diarrhea virus 1 | ORSV | Ovine respiratory syncytial virus |
| CCHFV | Crimean-Congo hemorrhagic fever virus | PDV | Penicillium discovirus |
| ChPV | Chaphamaparvovirus | PolB | DNA polymerase type B |
| CmcV1 | Chinese mitten crab virus 1 | ProPV | Protoparvovirus |
| Conn | Phage connector | PUUV | Puumala virus |
| CoPV | Copiparvovirus | PVBV | Palo verde broom virus |
| CTL | C-type lysozyme/alpha-lactalbumin family | RVA | Rotavirus A |
| CVV | Cache Valley virus | RVB | Rotavirus B |
| DependoPV | Dependoparvovirus | RVC | Rotavirus C |
| EncP | DNA encapsidation protein | RVFV | Rift Valley fever virus |
| Endo | Endolysin | SBV | Schmallenberg virus |
| EV | Enterovirus | SEOV | Seoul virus |
| FMV | Fig mosaic virus | SFTSV | Severe fever with thrombocytopenia syndrome virus |
| GBEEV | Ganda bee virus | SIMV | Simbu virus |
| GCFSV | Groundnut chlorotic fan-spot virus | TerL | Large terminase phage packaging protein |
| HbMV5 | Húběi myriapoda virus 5 | TetPV | Tetraparvovirus |
| HJRP | Holliday junction resolvase-like protein | TSWV | Tomato spotted wilt virus |
| HNH | HNH endonuclease | TuSV | Tulip streak virus |
| HOL | Holin | vcAAI50 | 50% average amino acid identity viral clusters |
| H-T adaptor | Head to tail adaptor | vcAAI90 | 90% average amino acid identity viral clusters |
| HTCP | Bacteriophage head to tail connecting protein | WhMV1 | Wǔhàn mosquito virus 1 |
| ICV | Influenza C virus | WhMV2 | Wǔhàn millipede virus 2 |
| IDV | Influenza D virus | WICV9 | Wēnlǐng crustacean virus 9 |

Table S2. Primer information used in this study.

| Virus | Primer^a^ | Sequence (5'-3') | Target length (bp) | Tm (℃) |
| --- | --- | --- | --- | --- |
| Bunya.un | JHTN-Of | AAAGAAACATGGATGGGTTTGG | 361 | 53.21 |
|  | JHTN-iF | TCAGAAACTGTTGCTGAACTTC |  | 52.7 |
|  | JHTN-R | ATGTCTGTCCATTGGTGAGC |  | 55.02 |
|  | DJN-F | TGTGTTGTAATGCAAGCACCA | 573 | 54.92 |
|  | DJN-oR | TGAAGAGTTTGGACCGGTGA |  | 56.06 |
|  | DJN-iR | ACTAGGTACAACAGCAGGCAC |  | 57 |
|  | GSN-oF | TTTCTTTGAATACCCGGCCA | 534 | 54.06 |
|  | GSN-oR | AGGCCATCTGCAAGTTTTTCA |  | 54.79 |
|  | GSN-iF | ATTGCTTGTTGCTGTTCACAT |  | 52.67 |
|  | GSN-iR | TTGCATCACAGTCAAGCAGT |  | 54.45 |
|  | HLWN-F | AATGTCACAATGTTGATTCATGCC | 179 | 53.72 |
|  | HLWN-oR | TCTCACATATGATTCGGCTGTT |  | 53.34 |
|  | HLWN-iR | ACACTAATTTCTCTTGCTCCCT |  | 53.05 |
|  | LZN-F | ATTTGGTTTCTGCTTTGGCG | 337 | 54.11 |
|  | LZN-oR | ATGAGAACAACCGAACCAGC |  | 53.34 |
|  | LZN-iR | TCAAATTTTCTCACGTTGGGC |  | 53.52 |
|  | SY-oF | TTTTCTGAACACGTGCACTG | 564 | 53.33 |
|  | SY-R | TGTCAAATTCTCGTCCTTGGT |  | 55.44 |
|  | SY-iF | TCAATTCACCGGGAGTCAAA |  | 53.68 |
|  | GLN-F | AGAGAGTTAAACTTGGGAAAGCA | 329 | 53.47 |
|  | GLN-oR | TTGCACAACATTCAGCCCAA |  | 55.31 |
|  | GLN-iR | TGATGAACATGCCAATATGCCT |  | 54.24 |
|  | WBY-F | TCTTCATCTTGTTTGAGGCCT | 439 | 53.50 |
|  | WBY-oR | TGATGTTGACAAACGAGCTGA |  | 55.31 |
|  | WBY-iR | AGAACAGTGGCTCAAGAGTG |  | 54.24 |
|  | XDN-F | ACCAAATGGAATGCTCACCA | 317 | 54.37 |
|  | XDN-oR | TCCATTCTTGCATTTGATCCGT |  | 54.33 |
|  | XDN-iR | ATCTGTTTGGGTTGGCTCGT |  | 57.01 |
|  | XN-F | CAGAAAAGAATGGACTGGTGAAA | 309 | 52.63 |
|  | XN-oR | TGTTCATACATTCCACTTTGGCT |  | 54.03 |
|  | XN-iR | ACCAATCTATGTGTGTTTGGGA |  | 53.55 |
|  | ZDN-oF | TGCAAAAATGAACGCCTCTC | 662 | 53.53 |
|  | ZDN-R | TTCAGCTGTCAAACTTGGCA |  | 54.67 |
|  | ZDN-iF | AATGGATTGAAAGCGCATTGA |  | 52.54 |
| Hepe.un | GS-oF | GCCAAGGAGTTAGCGCAATG | 351 | 59.90 |
|  | GS-oR | ATTCCAACCGCGAGGTTCTC |  | 60.39 |
|  | GS-iF | AAGCAGGCCAAGGAGTTAGC |  | 60.32 |
|  | GS-iR | ACAACCGCTCAATTGCATCC |  | 59.47 |
|  | NM-oF | GCTCAATGACATGTCCGGTC | 319 | 58.99 |
|  | NM-oR | TGTCCTCGTGGATCGTGAAC |  | 59.76 |
|  | NM-iF | CGAGGCGGACGTATCAGAAT |  | 59.69 |
|  | NM-iR | ATCAACTCACCTGCGTGGTC |  | 60.32 |
| FMDV | FMDV-F | TGTCAGACCTTCCTGAAGGACG | 621 | 59.96 |
|  | FMDV-R | CCTTTGTCGCTTTTGTCAGCTGG |  | 62.1 |
| PPRV | PPRV-oF | TTCAATGCTGGCTGCGTACAA | 476 | 61.15 |
|  | PPRV-iF | TCAGAGATGAGCCTAGTAGAG |  | 55.15 |
|  | PPRV-R | TTGTACGCAGCCAGCATTGAA |  | 61.15 |
| Nebovirus | NeV-oF | CCCGTACACTAGCAACCCTG | 595 | 58.35 |
|  | NeV-oR | AATGCTCTTGGTGAGGTGGG |  | 57.82 |
|  | NeV-iF | TTCGCACAGCCATACAGTCAT |  | 56.59 |
|  | NeV-iR | CGGGTCTTGAGGGTTTGACA |  | 57.61 |
| α-CoV | ACoV-F | TGTTGAACAGGTCTTGGCTG | 284 | 58.32 |
|  | ACoV-oR | CCGCTATAATAAAACCGCGACA |  | 59.20 |
|  | ACoV-iR | AAACACATGTCAAGCCAGCA |  | 58.32 |
| BoCoV | BCoV-oF | GTTGCGGCTATAACAAGCGG | 579 | 57.21 |
|  | BCoV-oR | TCAAAACTTGCGCGCATTCA |  | 55.94 |
|  | BCoV-iF | TGCTGTTTTAGTGTTGCGGC |  | 56.65 |
|  | BCoV-iR | AGGAACACCACGTGTAGCTG |  | 57.53 |
| EPV | PgVE-oF | ATGCCCTTTCCACTGGCTAC | 433 | 57.87 |
|  | PgVE-oR | AGCCTCCATAGCTGGTGAGA |  | 58.06 |
|  | PgVE-iF | GCAGGTTGCGCGTATACTTG |  | 57.14 |
|  | PgVE-iR | TCAACCACCACTTCGGCAAC |  | 58.59 |
| BVDV1 | BVDV1-oF | ATGCCATTGAGTCAGGGGTG | 432 | 57.89 |
|  | BVDV1-oR | GACAAGGCCTGATACCCCAC |  | 58.54 |
|  | BVDV1-iF | CCGGGTCAAAGGACTACCAC |  | 58.36 |
|  | BVDV1-iR | TCGGGCCAGTCCATACCTAA |  | 58.04 |
| ICV | IiF2-F | AGCAGGAGTTGGCGGTTATC | 446 | 60.11 |
|  | IiF2-R | GGGGAATGGTTGGAACAGGT |  | 59.89 |
| BRAV | BRAV-oF | GGAACAACTGTTGCAGGCTG | 775 | 57.41 |
|  | BRAV-oR | CACCCAACAGCGGAACCTAT |  | 57.76 |
|  | BRAV-iF | GGTGATGAACTCCCGAACGT |  | 57.58 |
|  | BRAV-iR | TCAGGTGTTCAACGGGTAGC |  | 57.58 |
| BRBV | BRBV-F | CCCACCTCAGACGAAGGCA | 533 | 61.58 |
|  | BRBV-R | CGAGGACAAGCGCATAACGA |  | 60.80 |
| ERAV | ERBV-oF | TGCTAATCAGCCACTGCCTC | 245 | 57.78 |
|  | ERBV-oR | CTCCACAAAAGGGCGGAAGA |  | 57.94 |
|  | ERBV-iF | CTGGAGCGTTTTCGCAAGTC |  | 57.28 |
|  | ERBV-iR | GCTAGGTCCGTGGTTGACAG |  | 58.54 |
| EV | EV-oF | TGTACGCCTGTTTTCCCCTC | 259 | 59.96 |
|  | EV-oR | CGCTCGGAGGTTGGGATTAG |  | 60.25 |
|  | EV-iF | GCGTAACGGTCAAGCACTTC |  | 59.84 |
|  | EV-iR | ACCATCCCAGCACAATAGGC |  | 60.11 |
| GHV | HV-oF | GTCGACTAAGGCCAGCAACT | 461 | 57.61 |
|  | HV-oR | ATTCCCTCCCTCCCTTTGGA |  | 58.24 |
|  | HV-iF | CCCCCATGAGTTGAAGCCTT |  | 57.9 |
|  | HV-iR | CTCCCTTTGGACGCCTATGG |  | 58.53 |
| Kobuvirus | KoV-oF | GTACCCCTGGAACACCATGG | 247 | 58.54 |
|  | KoV-oR | TGATTGCTCGTAGGTGTCCG |  | 57.31 |
|  | KoV-iF | TCATTCCCACCACGTGTACG |  | 57.54 |
|  | KoV-iR | GGGGTGTGCTTGTCGTAGAA |  | 57.58 |
| BTV | BToV-oF | CCCTACGCAACTACCACAGG | 344 | 58.35 |
|  | BToV-oR | GAACGCCAGGATGAAGTGGA |  | 57.68 |
|  | BToV-iF | GGGGCATTGTTGTTGACCAC |  | 57.52 |
|  | BToV-iR | CGCCTACGACGTGTATCCTC |  | 57.97 |
| GTV | GToV-oF | CTCACTGGGCTGGCTAACAA | 299 | 57.71 |
|  | GToV-oR | GAGGTTCCAGCAGATGCAGT |  | 57.74 |
|  | GToV-iF | CTGGACTGAACACCCTCGAC |  | 58.24 |
|  | GToV-iR | GTGGCAGCGCGTAAAGTATG |  | 57.14 |

a: oF, outer forward primer; iF, inner forward primer; cR, shared reverse primer; dF, shared forward primer; oR, outer reverse primer; iR, inner reverse primer.
